# Synthetic Cells and Molecules in Cellular Immunotherapy

**DOI:** 10.7150/ijbs.94346

**Published:** 2024-05-11

**Authors:** Haikun Lin, Chentao Li, Wanying Zhang, Boxiang Wu, Yanan Wang, Shimin Wang, Dongrui Wang, Xia Li, He Huang

**Affiliations:** 1 Bone Marrow Transplantation Center of The First Affiliated Hospital & Liangzhu Laboratory, Zhejiang University School of Medicine; 2 Institute of Hematology, Zhejiang University, Haining, China.; 3 Zhejiang Province Engineering Research Center for Stem Cell and Immunity Therapy, Haining, China.; 4 Zhejiang University-University of Edinburgh Institute, Zhejiang University School of Medicine, Zhejiang University, Haining, China.; 5 Bioscience and Biomedical Engineering Thrust, The Hong Kong University of Science and Technology (Guangzhou), Guangzhou, China.; 6 Department of Gastroenterology, Ruijin Hospital, Shanghai Jiao Tong University School of Medicine, Shanghai, China.

**Keywords:** cellular immunotherapy, CAR-T, synthetic cells, synthetic molecules, synthetic biomaterials

## Abstract

Cellular immunotherapy has emerged as an exciting strategy for cancer treatment, as it aims to enhance the body's immune response to tumor cells by engineering immune cells and designing synthetic molecules from scratch. Because of the cytotoxic nature, abundance in peripheral blood, and maturation of genetic engineering techniques, T cells have become the most commonly engineered immune cells to date. Represented by chimeric antigen receptor (CAR)-T therapy, T cell-based immunotherapy has revolutionized the clinical treatment of hematological malignancies. However, serious side effects and limited efficacy in solid tumors have hindered the clinical application of cellular immunotherapy. To address these limitations, various innovative strategies regarding synthetic cells and molecules have been developed. On one hand, some cytotoxic immune cells other than T cells have been engineered to explore the potential of targeted elimination of tumor cells, while some adjuvant cells have also been engineered to enhance the therapeutic effect. On the other hand, diverse synthetic cellular components and molecules are added to engineered immune cells to regulate their functions, promoting cytotoxic activity and restricting side effects. Moreover, novel bioactive materials such as hydrogels facilitating the delivery of therapeutic immune cells have also been applied to improve the efficacy of cellular immunotherapy. This review summarizes the innovative strategies of synthetic cells and molecules currently available in cellular immunotherapies, discusses the limitations, and provides insights into the next generation of cellular immunotherapies.

## Introduction

The immune system is an intricate network of cells, tissues, and organs that collaborate to protect the body from abnormal cells like tumor cells and harmful pathogens. Vast amounts of effort have been poured into developing strategies to leverage intrinsic immunity to combat various diseases, termed immunotherapies. Initially, strategies of immunotherapy mainly focus on editing individual genes and modifying antibodies and their derivatives. It has been gradually recognized that these strategies suffer from problems like insufficient affinity and immunogenicity, which make it hard to overcome existing therapeutic challenges 1. Immune cells are the major mediators and executants of immune functions, distributed around the body to supervise and eliminate any pathologic insults. Moreover, the immune cells are relatively easy to remove, engineer, and transfer back into a patient, making them significant candidates for immunotherapies 2. Therefore, cellular immunotherapies utilizing a diverse range of immune cells have drawn much attention and yielded exciting breakthroughs. Besides directly modifying cells, cellular immunotherapies also focus on designing synthetic molecules that can influence immune cell functions by affecting membrane receptors, transcription, cell death, etc.

Among the immune cells utilized by existing cellular immunotherapy research, T cell is the main engineered cell type due to their cytotoxicity, abundance in peripheral blood, and maturation of experimental engineering techniques. The major engineered T cells currently available include chimeric antigen receptor (CAR)-T and T cell receptor (TCR)-T. CAR-T cell is currently the most widely studied engineered immune cell in both basic research and clinical applications. CAR-T cells are generated from autologous T cells, which are genetically engineered via viral transduction to express the tumor antigen-specific CAR 3,4. The CAR recognizes tumor antigens and activates T cell cytotoxicity through the co-stimulatory domains in its structure. CAR-T therapy has revolutionized the clinical treatment of hematological malignancies, including acute myeloid leukemia (AML) multiple myeloma (MM), etc. Meanwhile, exciting results have also been yielded in research and clinical investigations utilizing CAR-T cells to treat other diseases, for example, liver fibrosis, acquired immune deficiency syndrome (AIDS), and systemic lupus erythematosus (SLE). 5-7. However, various challenges have hindered the efficacy and extended application of CAR-T therapy, including lack of proper antigens, T cell exhaustion, T cell carcinogenesis, etc. 8,9. Moreover, the efficacy of CAR-T therapy against solid tumors has been significantly limited by the complex tumor microenvironment (TME) within the solid tumor mass, which contains various hypoxia and immunosuppressive factors 10. Notably, a study reported the therapeutic effect of anti-CD19 CAR-T therapy can be inhibited by a single leukemic B cell mis-transduced with the CAR gene, which bound to the CD19 epitope on leukemic cells and masked the recognition by CAR-T cells. The clinical application of CAR-T is also commonly accompanied by serious systemic inflammation and organ dysfunction, termed cytokine release syndrome (CRS), which is caused by the hyperactivated immune system.

To address these challenges, diverse innovative synthetic strategies have been proposed, focusing on the two major aspects of cellular immunotherapies: synthetic cells and molecules. Strategies of synthetic cells have been focused on optimizing the structures of synthetic receptors on T cells (CAR and TCR). Diverse extracellular and intracellular domains have been added to the synthetic receptors on T cells, enhancing precise tumor antigen binding and intracellular signaling of cytotoxicity. Moreover, the therapeutic potentials of other engineered immune cells have also been investigated, including cytotoxic cells for targeted elimination of tumor cells and adjuvant cells for enhancing immune cell functions 2. Besides engineered immune cells, novel strategies of cellular immunotherapies also focus on designing synthetic molecules that form switches, which can control the expression and presentation of the CAR. Through molecular interactions and sensing chemical or physical changes in the environment around immune cells, the switches can precisely control the CAR expression, CAR degradation, and immune cell apoptosis, preventing overactivation. Furthermore, synthetic molecules that form logic gate circuits also exhibit promising results in CAR-T research, promoting precise elimination of tumor cells and preventing over-killing 11. Beyond synthetic cells and molecules, novel bioactive materials such as hydrogels that facilitate the precise delivery or in situ production of engineered immune cells have also been applied in research regarding cellular immunotherapies, generating positive results in preclinical models and holding promise for clinical applications 12,13. Additionally, it is also worth noting that many of these strategies have been developed to address specific issues in cellular immunotherapies, making them applicable to particular diseases.

This review summarizes the innovative strategies of synthetic cells and molecules currently developed in research regarding cellular immunotherapies. Advances in applying novel biomaterials in cellular immunotherapies are also reviewed. Existing limitations of these strategies are discussed, aiming to provide insights into the next generation of cellular immunotherapies.

## Synthetic Receptors for Programing T Cells

Engineered T cell therapies, represented by CAR-T and TCR-T immunotherapies, have shown great promise in clinical applications and have gained significant attention in recent years 3,4. Similar to CAR-T, TCR-T immunotherapy also uses synthetic biology approaches to optimize natural TCR structures to enhance tumor targeting and related cytotoxicity. Although both are engineered T cells, the two T cell immunotherapies produce distinct activation signals and face different limitations that require different synthetic strategies to overcome. Currently, the structural design of chimeric proteins has been one of the main strategies to improve the efficacy of T cell immunotherapies. The design of chimeric receptors is full of possibilities, even the traditionally preserved CD3ζ has been successfully replaced with other intracellular proximal T cell signaling molecules. Generally, the novel strategies of CAR or TCR structural design all aim to stably activate T cells while avoiding immune system over-activation and T cell exhaustion.

### Comparison between CAR-T and TCR-T immunotherapies

**Figure 1:**
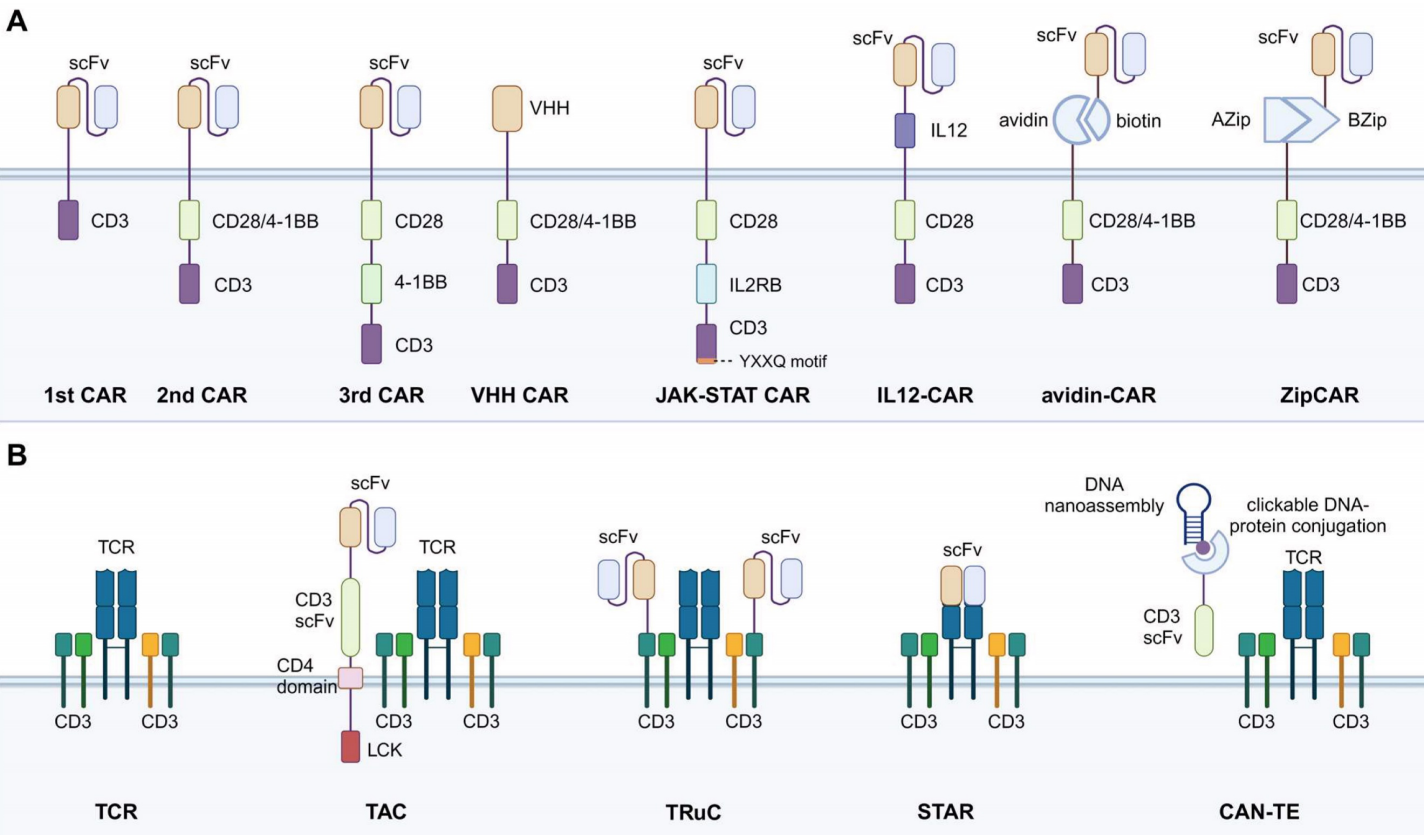
Overview of iterations of synthetic constructs of CAR and TCR. A. CAR construct iterations with diverse extracellular scFv domains and intracellular co-stimulatory domains. B. TCR construct iterations. CAR, chimeric antigen receptor. scFv, single-chain variable region fragment. VHH, single variable domain on a heavy chain. TAC, T cell antigen coupler. TRuC, T cell receptor fusion construct. STAR, synthetic T cell receptor, and antigen receptor. CAN-TE, chimeric antibody-nucleic acid T cell engager.

The structures of CAR and TCR are the most prominent differences between these two immunotherapies (Fig. 1). Basic CAR structure features the antibody single-chain variable region fragment (scFv) bound to the immunoreceptor tyrosine-based activation motif (ITAM) of CD3ζ. Co-stimulatory domains carrying a secondary signal (4-1BB or CD28) are also integrated into the intracellular part of CAR (Fig. 1) 14. By contrast, the TCR has more subunits in the receptor structure (from one to ten subunits), a larger ITAM, and more co-stimulatory receptors (CD3, CD4, CD28, etc.) 15. Recent studies have reported that the insertion of 4-1BB into the TCR structure can enhance the activation signals within T cells, but the location of 4-1BB relative to the CD3ζ domain (N-terminal or C-terminal) significantly affects TCR formation. The fusion of the 4-1BB domain to the N-terminal of CD3ζ could fail to constitute the TCR complex 16. As for CAR, the location preference for co-stimulatory signaling domain modification is reversed, reflecting another significant structural difference.

Mechanisms of antigen recognition mark another prominent difference between CAR-T and TCR-T (Table 1). CAR-T cells target tumor cell antigens by the specific scFv antibody in the CAR construct. TCR-T identifies tumor antigens by the natural binding affinity of TCR to the major histocompatibility complex (MHC) class Ⅰ protein presented by tumor cells. This TCR recognition mechanism provides the TCR-T with a wider variety of targets and the ability to identify target cells more specifically and efficiently 17. For target antigen screening, TCR-T can recognize tumor cells that express MHC-related proteins. For example, researchers recently screened an MHC-related protein 1 (MR1)-restricted novel T cell by using genome-wide CRISPR-Cas9 18. This TCR can mediate T cell killing of a variety of cancer cells, including those of lung, blood, colon, breast, bone, kidney, and cervical cancers by recognizing the monomorphic MR1 on the surface while remaining inert to non-cancerous cells. However, the TCR-based recognition mechanism is also a disadvantage of TCR-T compared to CAR-T. The binding of TCR to MHC class Ⅰ protein is highly specific, requiring specific TCR isoforms recognizing homologous MHC isoforms. Before the TCR engineering, specific TCR isoforms that pair with homologous MHC should be screened out first, enhancing the time and cost, which significantly hinder the clinical application of TCR-T. Moreover, various tumor cells have been found to escape TCR-mediated cytotoxicity by modulating the expression of MHC class Ⅰ proteins, which further limits the application of TCR-T 19.

The killing efficacy of TCR-T is not as strong as CAR-T (Table 1). CAR structure activates the original killing mechanism of the T cells through CD3ζ and other functional signals 20,21. CAR-T cells present a faster killing function and quickly metastasize to the next tumor target (sequential killing), contrasting to the long-lasting signaling and killing of TCR-T cells. TCR-T cells target antigens by forming an immunosynaptic (IS) structure, where TCR presents ring regions with peripheral lymphocyte function-associated antigen 1 (LFA-1). The CAR-binding to antigen shows diffuse LFA-1 distribution without ring regions 22. LFA-1 is a mechanosensitive adhesion receptor regulating T cell effector function by mechanically promoting T cell adhesion to target cells 23. Thus, TCR initiates a slower but longer-lasting signal than CAR. Furthermore, as the CAR is specifically designed for tumor antigens, the risk of autoimmunity is reduced compared to TCR. The MHC-dependent nature of TCR makes it potential to have higher risks of off-target toxicity and autoimmunity 24.

### Iterations of CAR Structure

The current structure of CAR has generally gone through four generations as the chimeric protein structures have been increasingly studied. The scFv antibodies are commonly used in CAR-T cells to bind to specific cell surface antigens, activating downstream signaling for cytotoxicity. From the view of technological development, CAR structures continue to be innovated, as advances in transduction technology have allowed more complex CAR structures to be implemented in cells. Innovative synthetic strategies that engineer the antigen-binding scFv and incorporate extra signaling domains into CAR structure are summarized below 25,26.

#### CAR with No Co-stimulatory Signal (the 1st CAR)

All CARs, including first-, second-, and third-generation CARs, are equipped with hinges that connect scFv to intracellular signaling structural domains. The hinge is usually derived from the CD8α glycoprotein or IgG constant domain. CD8α hinges can pass through the cell membrane via their hydrophobic transmembrane structural domains. The first-generation CARs did not contain co-stimulatory domains and their transmembrane structural domains were post-connected to the ITAM of CD3ζ (Fig. 1) 21,27. Naïve T cells need to transform into effector T cells stimulated by the first stimulatory signal generated by TCR pathway activation and activated by the second stimulatory signal provided by CD28/B7 pathway activation to have the ability to kill tumors. Therefore, this generation of CAR-T suffers from inefficient transformation caused by insufficient co-stimulatory signals, which have been resolved by second-generation CAR-T.

#### CAR with One Co-stimulatory Signal (the 2nd CAR)

The second-generation CARs have the main CAR structure currently applied in available CAR-T products and treatments. A co-stimulatory domain is added to the intracellular structural region of the second-generation CARs, allowing CAR-T cells to activate both stimulatory and co-stimulatory signals 28, and in turn, enhance the transformation efficiency. Co-stimulatory molecules usually contain CD28 or 4-1BB fused with intracellular signaling domains derived from the CD3ε sequence (Fig. 1). CD28 and 4-1BB are the two most used co-stimulatory factors and share the same signaling pathway. CD28 CAR-T has a stronger cellular effector function than 4-1BB CAR-T, as CD28 activation causes significantly higher levels of phosphorylation and produces higher levels of effector molecules (GZMB, TNF, IFN-γ, IL-2, etc.). However, CD28 CAR-T also expresses higher levels of depletion markers (immune checkpoint molecules: PD-1, Lag-3, Tim-3, etc.) and has a shorter retention time 29.

#### CAR with Two Co-stimulatory Signals (the 3rd CAR)

The structure of third-generation CAR-T cells is similar to the second-generation, but third-generation CAR-T cells use lentivirus as a transduction vector, which can carry larger gene fragments into T lymphocytes. Therefore, the third-generation CAR intracellular segment often contains 2 or more co-stimulatory signals in the ITAM region (Fig. 1). In theory, third-generation CAR-T cells should have stronger activation and killing ability compared with second-generation CAR-T cells. However, studies have shown that the cytotoxicity of third-generation CAR-T cells is similar to that of second-generation CAR-T. Therefore, the simple addition of a co-stimulatory molecule does not necessarily enhance the efficacy. The possible explanation for this is that the activation signal generated by one co-stimulatory molecule already reaches the threshold of T cell activation. The simple addition of extra ITAM domains will not further enhance the activation effect of CAR.

#### CAR with Nanobodies

Nanobodies refer to the single variable domain on a heavy chain (VHH) of antibodies. Compared to the scFv antibodies, nanobodies have a smaller size but similar binding affinity, making them potential to replace the scFv in the CAR construct (Fig. 1) 1. Research has applied the VHH nanobodies as the targeting domain of CAR and found that compared to scFv-CAR, the stability of VHH nanobodies was higher and the immunogenicity was lower 30. Moreover, the VHH nanobodies have longer complementary-determining region 3 (CDR3), a region that determines the antigen-binding activities of the antibodies. As a result, the antigen-recognition spectrum of VHH nanobodies is broader than the scFv, enabling the CAR-T cell to target more types of tumor cells 31. The compact size of VHH nanobodies also offers exceptional prospects for optimizing the hinge and transmembrane structural domains of CAR, such as the IgG4 hinge with CD28 transmembrane domain to improve CAR-T membrane-distal epitope recognition 32.

#### CAR with Integrated Cytokine Signal

In addition to stimulatory and co-stimulatory signals provided by the CD28/B7 pathway, as discussed above, cytokine involvement is also required for full CAR-T cell activation 33. To take advantage of cytokine signaling, some research has integrated the functional parts of cytokines into the CAR construct. For instance, Hinrich et al. inserted a functionally active single-chain p40-p35 variant of IL-12 into the extracellular portion of the CAR (Fig. 1) 34. The use of IL-12-CAR converted cytotoxic T cells to a CD8^+^ CD56^+^ CD62L^high^ phenotype, downregulated Th2 genes, and upregulated NK and cytokine-induced killer (CIK) cell-related genes. Functionally, IL-12-CAR-T cells also acquire antigen-independent lytic activity, making antigen-heterogeneous tumors accessible to IL-12-CAR-T cell attack. Technically, IL-12-CAR has more transfectable properties compared to T cells redirected for universal cytokine killing (TRUCK, a strategy discussed below) but is less capable of mobilizing other immune cells for killing because its IL-12 is not released into the tumor environment. This structure allows the transformation of CAR-T cells into NK-like phenotypes, adopting a cytotoxicity mechanism similar to NK and CIK cells.

In addition to directly integrating cytokines into the CAR construct, there are some studies fusing the intracellular signaling factors of cytokines into the CAR construct. For example, Naoto Hirano developed a novel CAR construct containing a truncated cytoplasmic domain of IL-2Rβ and a STAT3-binding YXXQ motif accompanied by CD3z and CD28 structural domains (28-ΔIL2RB-z (YXXQ)). The extra domains confer the CAR structure with the ability to induce IL-2 signaling, which mainly involves JAK-STAT3/5 pathway activation (Fig. 1). In vitro experiments exhibited promoted CAR-T cell proliferation and reduced terminal differentiation. Moreover, compared to the second-generation CAR-T cells, the 28-ΔIL2RB-z (YXXQ) CAR-T cells also showed greater persistence in vivo and anti-tumor effects in either liquid or solid tumor models 14. Furthermore, the concept of specifically utilizing cytokine signaling with CAR improves the specificity of cytokine functions, avoiding cytokine over-release, which could lead to severe inflammatory toxicity. Other than the cytokines discussed here, various other cytokines have also been applied in immunotherapies, as summarized in Table 2.

#### Split, Universal, and Programmable (SUPRA) CAR

The construct of SUPRA CAR is inspired by the concept of bispecific T cell engager (BiTE) therapy, which utilizes the bispecific binding affinities of special antibodies. The BiTE can identify and bind to specific tumor antigens on the one end and bind to CD3 on T cells on the other end 35. Based on this, research has applied bispecific antibodies in CAR design. The intracellular and extracellular domains of a normal CAR construct are split in SUPRA CAR, with each fusing with mediator proteins that can bind to each other. For example, the avidin-CAR is based on avidin-biotin, and the ZipCAR is based on AZip-BZip (Fig. 1) 36,37. This SUPRA CAR structure has advantages in universal tumor targeting and killing, as the CAR-T cells do not need to be specifically engineered to target tumor antigens. The tumor targeting is achieved by injecting different mediator proteins conjugated with tumor-specific scFv, which is easier and cheaper than T cell engineering. However, the mediator proteins investigated have shown shorter circulating half-life due to the elimination of the crystallizable Fc segment of the antibody. Serious risks of side effects including CRS and central nervous toxicity remain insurmountable problems even with a dose-escalation administration 38.

### Iterations of TCR Structure

While diverse synthetic strategies concerning CAR-T cell design have been proposed, few studies have explored the modification of the signaling structural domain of the TCR complex to enhance the function of TCR-T cells. Many studies on TCR-T cells have attempted to identify TCRα/β sequences specific for tumor antigens or to increase their affinity. The extracellular structural domains of the TCR complex have been modified by complementing or replacing them with variable structural domains from antibodies, as discussed below.

#### T cell Antigen Coupler (TAC)

Similar to CAR, TAC is also a synthetic chimeric receptor transduced into the T cells. The difference from CAR is that TAC only takes the role of targeting tumor antigens and utilizes endogenous TCR signaling for cytotoxic abilities. TAC contains three components: an extracellular antigen-binding scFv, an extracellular anti-CD3 scFv, and a transmembrane CD4 co-receptor domain (Fig. 1) 39. TAC couples to the endogenous TCR on the T cell membrane by the anti-CD3 scFv. The tumor antigen-binding scFv facilitates specific targeting of tumor cells and activates the TCR-mediated cytotoxicity via the CD4 co-receptor domain. Through this construct, the T cell-mediated cytotoxicity can be redirected into a TCR-dependent, while MHC-independent manner. TAC-engineered T cells have generated more robust and efficient anti-tumor responses in xenograft models and in vitro, while exhibiting reduced over-release of cytokines and toxicity, compared to CAR-T cells. Furthermore, the TAC-engineered T cells also showed increased anti-tumor efficacy and faster infiltration in a solid tumor model bearing HER2, compared to CAR-T cells 40.

#### T cell Receptor Fusion Construct (TRuC)

TRuCs are antibody-binding structural domains that fuse scFv to TCR subunits and can effectively reprogram the intact TCR complex to recognize tumor surface antigens 41. In contrast to CAR, TRuC integrates the antigen-binding mode of CAR as a functional component of the TCR complex (Fig. 1). In an in vitro model, TRuC-T cells demonstrated a tumor cell-killing efficiency comparable to second-generation CAR-T cells, even though the TRuC does not possess co-stimulatory domains like CD28 or 4-1BB. Notably, TRuC-T cells exhibited considerably lower cytokine release. In addition, TRuC-T cells exhibited powerful anti-tumor activity in either liquid or solid tumor xenograft models. In some models, TRuC-T cells were even more effective than the corresponding CAR-T cells.

#### Synthetic T Cell Receptor and Antigen Receptor (STAR)

STAR is a two-chain structural complex obtained by fusing scFv with the constant alpha and beta chain regions of TCR (Fig. 1) 42. Compared to TRuC, STAR discards the TCR antigen-binding structural domain and thus has a smaller protein bulk. STAR-T cells exhibit a specific killing function comparable to that of CD28-CAR-T cells while maintaining the same survival and proliferation capacity as 4-1BB-CAR-T cells. In animal models, STAR-T cells outperformed CAR-T cells in controlling a variety of mouse tumors 42. They exhibited better efficacy and anti-depletion effects than CAR-T, showing promise for clinical applications. In particular, the STAR gene has a smaller gene length and is easier to transduction into T cells.

#### Chimeric Antibody-nucleic Acid T cell Engager (CAN-TE)

CAN-TE, another modular, intelligent, and tunable complex based on synthetic biology, has shown promising applications through DNA recognition mechanisms such as precise complementary pairing ability, sequence programmability, structural designability, and unique mechanical properties 43. The CAN-TE complex contains two functional fractions: a DNA nanoassembly that can recognize and bind to tumor-specific antigens, and an anti-CD3 scFv that engage with CD3 co-receptors in the TCR complex (Fig. 1). These two fractions are conjugated to each other via a dense core vesicle (DCV) endonuclease-supported click-type DNA protein. The tumor-targeting DNA is chemically synthesized based on an in vitro single-stranded oligonucleotide library (SELEX). CAN-TE utilizes the TCR-mediated cytotoxicity in an MHC-independent manner, avoiding the restriction of TCR-T posed by TCR isoforms. In addition, the construct of CAN-TE can also be applied to identify multiple targets, mediating the AND logic gate circuits, which are achieved by the dynamic tumor-targeting DNA sequences in SELEX. However, it remains to be investigated whether the binding spectrum of nucleic acids in SELEX contains specific tumor antigens and whether the binding affinity is ideal.

Overall, the novel synthetic strategies in optimizing CAR and TCR constructs have shown great promise in in vitro and in vivo preclinical studies, and further research examining their clinical applications is valuable. It's worth noting that many of these strategies have been developed to address specific issues, making them applicable to particular diseases or contexts. Moreover, the selected antigen, which is targeted by CAR or TCR, also impacts the treatment outcome. For instance, in the case of anti-FAP CAR-T used to treat cardiac fibrosis in a mouse model, no notable adverse effects were observed 44, which is significantly different from anti-BCMA and anti-CD19 CAR-T in multiple myeloma 45. Therefore, it is difficult to determine a universally effective design for T cell immunotherapy.

## Engineered Cellular Immunotherapy Beyond Conventional T cells

**Figure 2:**
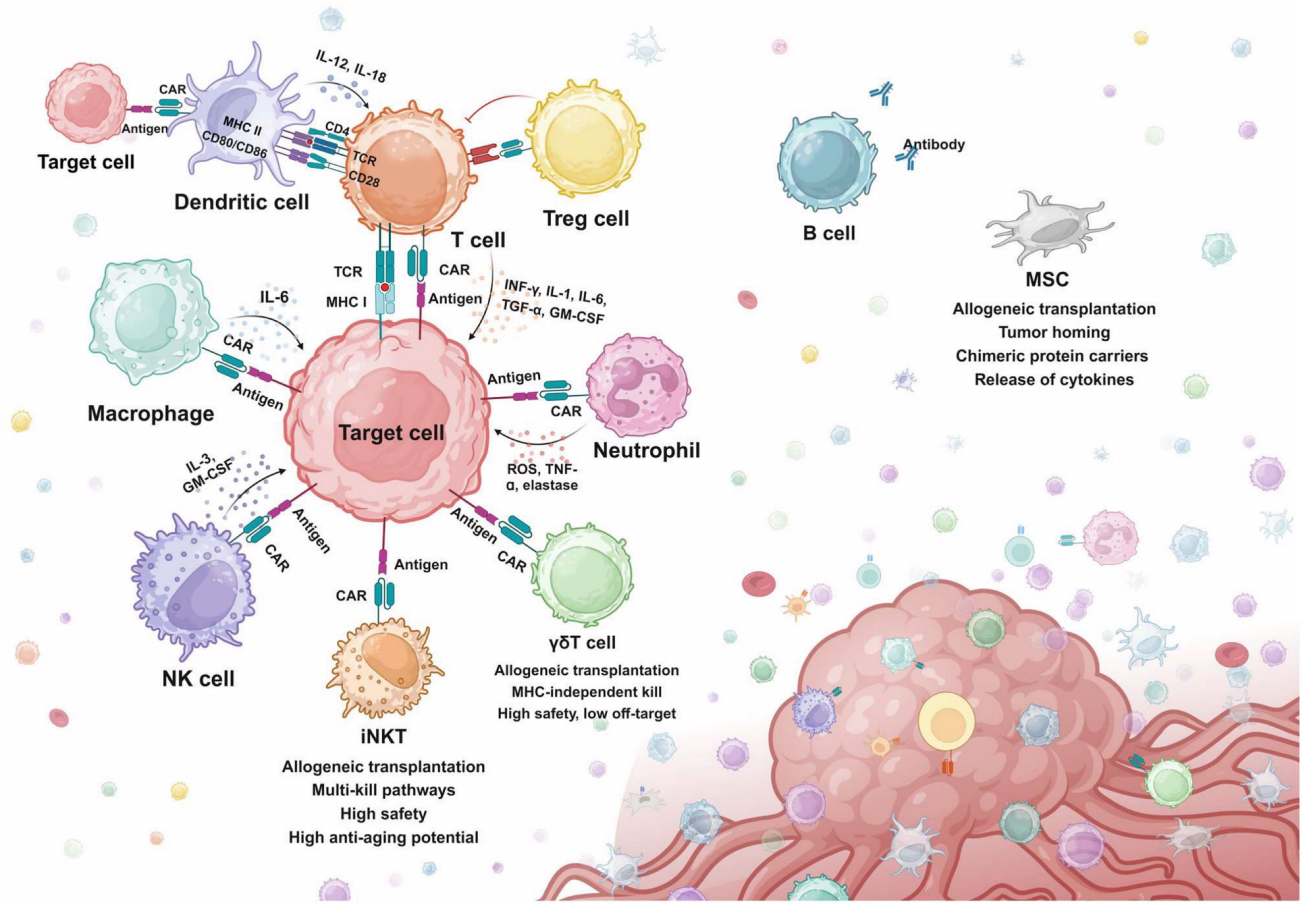
Overview of synthetic killing and adjuvant systems. iNKT, Invariant natural killer T cell. MHC, major histocompatibility complex. MSC, mesenchymal stem cell. NK cell, natural killer cell. Treg cell, regulatory T cell.

As discussed above, T cell-based cellular immunotherapies have been extensively studied and various strategies have been developed for resolving specific issues. However, T cell immunotherapies still face challenges of tumor relapse, which failed to be overcome by screening for alternative antigens, structure optimization, and multi-target CAR design 46-48. Moreover, the efficacy of both CAR-T and TCR-T has been significantly constrained by problems like tumor heterogeneity, antigen escape, off-target toxicity, and immunosuppressive TME in the treatment of solid tumors 49. Therefore, many studies have shifted attention to exploring the feasibility of engineering other immune cell types for treatment purposes. Attempts have been made to engineer other cytotoxic cells for tumor eradication, which exhibit better safety, stability, and tumor-infiltrating abilities in preclinical models. Besides cytotoxic cells, other adjuvant immune cells have also been engineered to assist or improve the efficacy of CAR-T therapy. In this section, advances in engineering immune cells beyond T cells are reviewed (Fig. 2).

### Engineered Cytotoxic Cells

#### Natural Killer (NK) Cells

NK cells are the innate immune killers that recognize and eradicate tumor cells via a special mechanism termed “missing-self”. Contrary to the TCR recognition mechanism, NK cells are activated via a balance between activating signals by tumor antigens and inhibitory signals by MHC class Ⅰ proteins. Several tumor cells have been found to evade T cell recognition by downregulating the expression of MHC class Ⅰ on the surface, while NK cells can recognize the missed MHC protein and trigger NK-mediated cytotoxicity 50. Infused NK cells for cancer treatments were not commonly associated with CRS, which potentially demonstrates a better safety of NK over T. Moreover, unlike T cells, the NK cells do not express TCR, which can lead to significant graft-versus-host disease (GVHD) when encountering MHC mismatched hosts 51,52.

Based on the advantages of NK cells, various studies have been engineering NK cells with chimeric proteins to construct CAR-NK (Fig. 2). Synthetic strategies in designing CAR-NK focus on enhancing the anti-tumor activity by genetically modifying NK cells to promote activation, expansion, and cytotoxicity. Compared to CAR-T cells, CAR-NK cells exhibited comparable killing efficiency in both in vitro co-culture and in vivo mice models, while showing a great improvement in safety and versatility 53-55. In a most recent phase 1/2 clinical trial, patients receiving CD19-CAR-NK exhibited 48% overall response rates and 68% 1-year overall survival, with no obvious toxicities like CRS or GVHD observed 56. However, adult peripheral blood NK cells are relatively resistant to retroviral and lentiviral transduction and exhibit poor persistence in the absence of high levels of IL-2 or IL-15. To circumvent this, the NK cells in unrelated cord blood were transduced to express a CD19-targeting CAR, and a transgene coding for IL-15, which showed outstanding efficacy in a xenograft lymphoma murine model and clinical trials 56,57.

CAR-NK also exhibits promising off-the-shelf potentials according to a recent study constructing CAR libraries containing diverse scFvs that recognize a vast range of tumor antigens. CAR-NK engineered with clones from the CAR library completely eradicated epithelial tumor cells in mice models with murine xenografts and patient-derived xenografts, showing great promise for clinical applications 58. However, as more suitable tumor antigens and corresponding scFvs are yet to be discovered for many tumors, CAR-NK libraries still have a long way to go in the clinic. Furthermore, the off-the-shelf potential of CAR-NK has also been validated by clinical reports using allogeneic cord blood-derived CAR-NK for lymphoma patients 53,59.

#### Invariant Natural Killer T (iNKT) Cells

iNKT cells have been investigated for CAR engineering. iNKT cells are a specialized small subset of T cells that display both innate and adaptive immune features. They express TCR on the surface, but they only recognize lipid antigens presented by monomorphic MHC class 1-like molecules. iNKT cells can also be rapidly stimulated by various cytokines, which is a feature of innate immune cells like NK cells 60. Activated iNKT cells can mediate effective anti-tumor responses either through direct tumor lysing or indirectly by secreting cytokines to mobilize other immune cells in the malignant environment 61. iNKT cells used for CAR engineering are mainly isolated from the peripheral blood. Anti-GD2 CAR-iNKT with IL-15 co-expression exhibited good killing efficacy in neuroblastoma cell models. Further in vivo experiments in mice bearing neuroblastoma xenografts demonstrated promising persistence, mobilization to targets, and tumor killing 62. Encouraging evidence has arisen from the interim results of the first-in-human phase 1 CAR-iNKT for neuroblastoma, showing sufficient expansion and anti-tumor activity after infusion 63. Interestingly, a study reported that tissue-resident iNKT exhibited senolytic efficacy when cultured with senescent cells in vitro 64. The potential intrinsic senolytic ability of iNKT cells could be enhanced by innovative CAR designs.

#### Gamma Delta (γδ) T Cells

γδ T cell is also a small subset of T cells, linking innate and adaptive immune responses. γδ T cells can be triggered in an MHC-independent manner and directly kill tumor cells via various strategies, including NK-like cytotoxicity mechanism, antibody-dependent cell-mediated cytotoxicity (ADCC) effects, and secreting effector cytokines (e.g. IFN-γ, TNF-α). γδ T also mediates the indirect killing of tumor cells by acting as antigen-presenting cells (APCs), presenting antigens to and activating αβ T, B, NK, and DC cells. At the same time, by sensing stress signals specifically expressed by cancer cells, γδ TCR can distinguish cancer cells from healthy cells. That is, it is completely safe for healthy cells 65,66 These characteristics make γδ T cells a potential platform for CAR engineering. Studies have attempted to transduce autologous γδ T cells with retroviral vectors containing anti-GD2 or anti-CD19 CARs 67,68. Promising results were generated in these studies in tumor cell lines and in vivo xenograft murine models, which paved the feasibility of CAR-γδ T for clinical applications. Clinical trials using anti-CD19 CAR-γδ T for B cell malignancies are in process 65.

#### Macrophage

It is well known that CAR-T has encountered obstacles in attacking solid tumors. In the TME, macrophages are the innate immune cells with the highest infiltration rate. Because macrophages can infiltrate solid tumor tissue and interact with almost all cellular components of the TME, CAR-macrophages appear to have an opportunity to attack solid tumors. The first-generation CAR-macrophage transduces pluripotent stem cells (iPSCs)-derived macrophages a CAR containing the CD3ζ activating domain. The CAR-macrophage exhibited promising results in tumor cell models, like enhanced phagocytosis of tumor cells and in vivo anticancer cell activity 69. The addition of a tandem PI3K recruitment structural domain to CAR-Phagocytic intracellular signaling further enhances tumor phagocytosis. This targeted phagocytosis resulted in greater in vitro tumor control than that induced by unmodified macrophages 70. Recently, a second generation of CAR-macrophage was proposed, which added a toll-like receptor 4 intracellular toll/IL-1R (TIR) domain to the CAR construct. Compared to the first generation CAR-macrophage, the TIR-containing CAR-macrophage exhibited markedly improved antitumor effects in both in vivo and in vitro mice models 71.

However, macrophages are more resistant to transduction compared to T cells. Current research regarding CAR-macrophage mainly obtains autologous macrophages from iPSCs, which raises the engineering time and cost. Moreover, CAR-macrophage gene transfer uses viral transduction, which may induce insertional mutations that have uncontrollable effects on therapy 72. Most importantly, it is well acknowledged that macrophages are the major cell mediators of inflammatory responses, significantly contributing to the CRS following CAR-T therapy. Thus, CAR-macrophages may also lead to serious inflammatory responses. Therefore, the clinical application potential of CAR-macrophage should be evaluated with caution in future studies.

### Engineered Adjuvant Cells

#### Dendritic Cells (DCs)

DCs are APCs with the capability of efficiently processing and superficially presenting antigens to activate the adaptive immune system (Fig. 2). They also perform uniquely when interacting with and regulating the function of multiple immune cell subsets in the lymph nodes, including B cells, NK cells, phagocytes, CD4+ and CD8+ T cells 73. Because of these unique characteristics, DC has been applied in immunotherapies to increase immune responses mediated by drugs and immune cells. DC vaccines are the typical application of DCs in immunotherapies and have been applied in several clinical trials to increase the number and function of tumor-specific T cells 74. The success of DC-based vaccines suggests that DCs isolated and amplified in vitro could enhance T cell-mediated cytotoxicity in vivo. Based on this, attempts have been made to design CAR-DC to express the same CAR with CAR-T and assist CAR-T cytotoxicity. Results from a pioneer study showed that combining anti-CD33 CAR-DC with anti-CD33 CAR-T cells enhanced CAR-T cell function and cytotoxicity compared to the results obtained with CAR-T cells alone, resulting in improved tumor control in AML xenograft mouse models 75. These results verified the feasibility of engineering DCs with CAR for improving antitumor responses. However, as a therapy that indirectly promotes tumor killing, CAR-DC is similar to previous DC-CIK therapies in that its specificity may not be ideal 76.

#### Regulatory T Cells (Tregs)

Treg cell is another subset of T cell which plays a vital role in regulating the body's immune response by maintaining self-tolerance, preventing excessive activation of immune responses, and also contributing to the tumor evasion of immune surveillance (Fig. 2). Due to the unique immunosuppressive functions of Tregs, the engineering strategies regarding Tregs mainly aim to target disorders generated by immune over-activation, such as autoimmune diseases, GVHD, and inflammatory disorders 77. In terms of CAR-Treg, Tregs are engineered to accumulate in vulnerable tissues to suppress local immune activities. The early attempts to engineer Tregs involve transducing anti-2,4,6-trinitrophenol (anti-TNP) CAR and anti-carcinoembryonic antigen (anti-CEA) CAR into murine peripheral blood-derived Tregs 78,79. Both TNP and CEA are correlated with the progression of colon inflammation (colitis). These promising results promoted the further research of CAR-Tregs in patients, but no consistent benefits have been generated. Other than autoimmune and inflammation disorders, CAR-Tregs have also been applied in GVHD. Unlike most autoimmune diseases, there are very clear targets in transplantation, namely HLA molecules. In 2016, HLA-A2 CAR-Treg cells were first reported, and studies demonstrated that HLA-A2^-^ CAR-Treg cells inhibited effector T cell proliferation and blocked HLA-A2^+^ PBMC-mediated GvHD in an immunodeficient NSG mouse model 77,80.

#### Neutrophils

Neutrophils are the most abundant leukocyte circulating in the body, and they can infiltrate and accumulate in various types of solid tumors (Fig. 2). There has been an extending body of studies showing that tumor-associated neutrophils (TANs) have both anti-tumor (N1) and pro-tumor (N2) types 81. Neutrophils have been engineered and applied in cell-mediated drug delivery due to their high motility in the blood, ability to physiological barriers, and intrinsic phagocytotic ability. However, the efficacy is limited because neutrophils are short-lived with a half-life of a couple of hours or days and they are resistant to gene editing 82. Engineering human pluripotent stem cells (hPSCs) and inducing the differentiation into CAR-neutrophils is therefore an effective alternative method. A study engineered hPSCs with anti-glioblastoma (GBM) CAR by CRISPR/Cas9 and then induced the differentiation into CAR-neutrophils. In the mice models, the anti-GBM CAR-neutrophils efficiently delivered and released TME-responsive nanodrugs to the GBM microenvironment without triggering any inflammation. Moreover, hPSCs-induced CAR-neutrophils also sustain the N1 phenotype, exhibiting direct tumor-killing activities as well as increased TNF-ɑ secretion and ROS (Reactive Oxygen Species) generation in an in vitro mimicked tumor immunosuppressive microenvironment 83. However, it is poorly understood how distinct types of TANs form. Engineered neutrophils may develop an N2 pro-tumor phenotype, which can be devastating 81.

#### B Cells

B cells secrete specific antibodies and present antigens to CD4+ T cells (Fig. 2). Antibodies are being widely used to treat diseases ranging from cancer to autoimmunity. Various engineered B cell strategies have been proposed and validated in animal models. For example, a study utilized CRISPR/Cas9 to engineer the endogenous antibody-expressing region in primary B cells. These B cells specifically expressed antibodies against respiratory syncytial virus (RSV) and effectively protected RSV-infected mice 84. B cell receptors (BCRs) have also been engineered to target specific tumor antigens, which accelerates and enhances the production of tumor-specific antibodies upon antigen recognition 85.

#### Mesenchymal Stem Cells (MSCs)

MSCs are widely available (common placenta, umbilical cord, fat, bone marrow, etc.), easy to obtain, ready to proliferate, and inherently low in immunogenicity. These advantageous characteristics contribute to the application in augmenting the efficacy of CAR-based immunotherapy (Fig. 2) 86. For example, MSC delivery of oncolytic immunotherapy has been proven to improve CAR-T cell anti-tumor activity 87. Recently, an MSC-based strategy to achieve anti-tumor universal cell therapy through antigen depletion has been proposed. MSCs carrying anti-CD19 anergic CARs (scFvs) were shown to be effective in initiating apoptosis of target cells in two leukemia cell lines, SEM and REH 88. Considering the absence of side effects like CRS, this seems to offer a new engineered anti-tumor strategy. In addition, as a cell that can be applied allogenically, the possibility exists that MSC can be coupled with CAR-T. A previous review has looked in detail at the possibilities of MSC in CAR-T for the treatment of several solid tumors 86.

The tumor homing ability of MSC as well as targeted modifications allow MSC to precisly migrate into tumor tissues. Thus, MSCs can be used as vectors for the delivery of therapeutic products like transgenic immunomodulators and bioactive proteins (supportive cytokines) to tumor tissue. This leads to a shift in the TME from an initially suppressed state to an immune-stimulating environment 89. Engineered MSC releases IL-7 and IL-12 in the TME 90. IL-7 enhances the expansion of T cells and maintains the memory cell function of T cells. MSC expressing IL-12 inhibited the metastasis of tumor cells. IL-12 induces protective Th1 responses and prevents Th2 polarization of T cells. The metastasis of tumor cells is also inhibited by MSC-derived IL-2 91.

## Synthetic Molecules for Precise Regulation of Cellular Immunotherapies

### Switches for Regulating Cell Activation

While cellular immunotherapies like CAR-T therapy have achieved great success in cancer treatment, they are still hampered by severe side effects including CRS and neurotoxicity, which are highly correlated to the hyperactivated interactions between CAR-T cells, tumor cells, and endogenous immune cells. The number of CARs presented on the surface of T cells is a key indicator to evaluate the anti-cancer activity and potential toxicity. Hence, it is essential to develop novel synthetic strategies to restrict immune activities by regulating CAR expression 92. Strategies using synthetic molecules to construct switches that control the expression and presentation of CAR have gained much attention and progress. A wide range of synthetic molecules is included, such as extracellular antibodies, intracellular enzymes, chemical compounds, and transcription factors. By sensing specific signals, these molecules form switches that precisely control the “on” and “off” of CAR-T actions 93-96.

#### Molecular Switch

Molecular switches in CAR-T therapy refer to switches formed and activated by small molecules/drugs. Based on the functioning pattern, proposed molecular switches for CAR-T therapies to date can be characterized into “on” and “off” switches.

**Figure 3:**
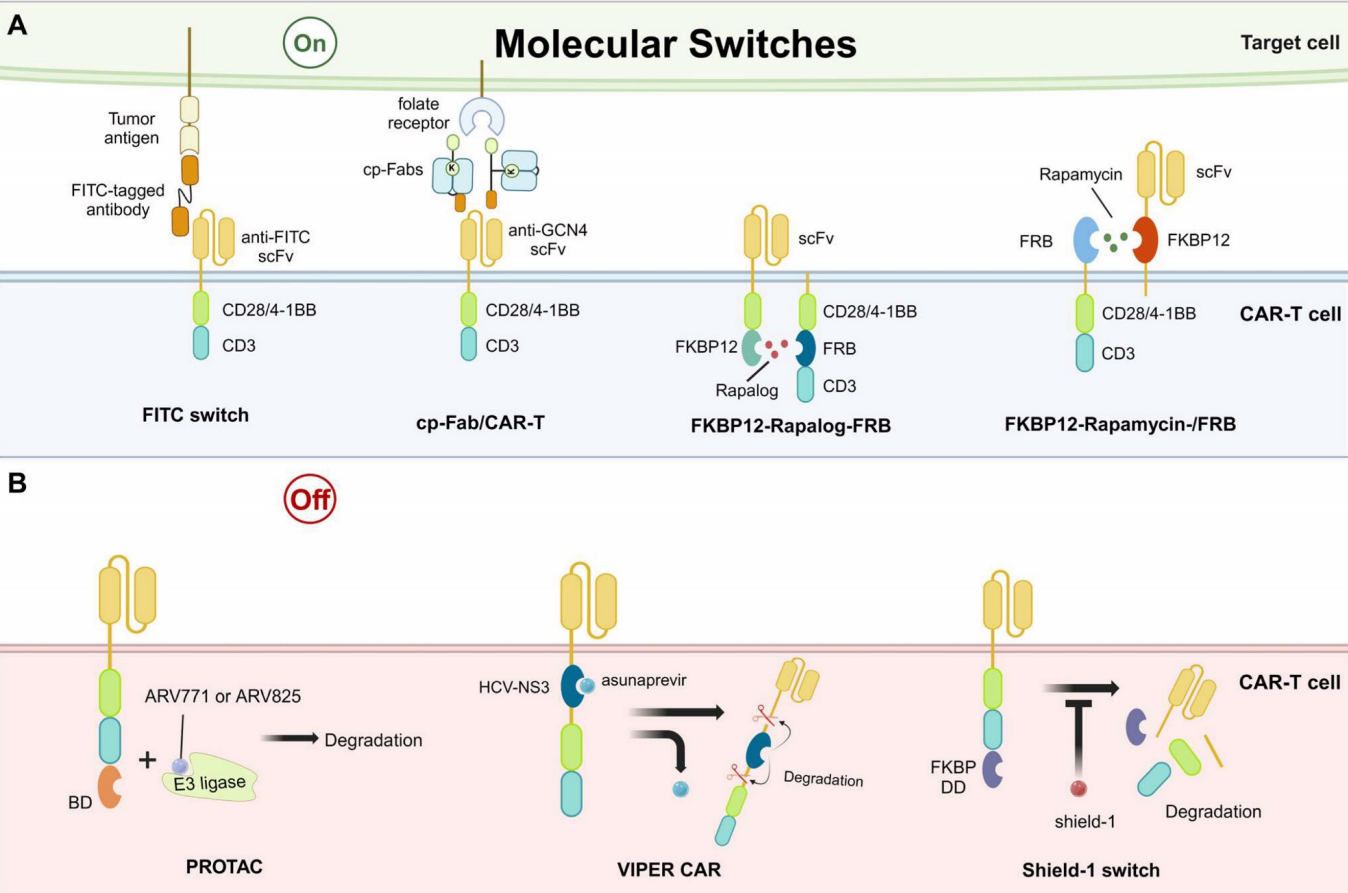
Overview of synthetic molecular switches applied in CAR-based killing systems. A. Examples of designing “on” molecular switches, which can initiate CAR-mediated killing. B. Examples of designing “Off” molecular switches, which can abrogate CAR-mediated killing by degrading the CAR structure. FITC, fluorescein isothiocyanate. cp-Fab, chemically programmed antibody fragment. FKBP, FK506-binding protein. FRB, FKBP12-rapamycin binding domain. BD, bromodomain. HCV-NS3, hepatitis C virus NS3 protease. PROTAC, protein hydrolysis targeted chimera. VIPER CAR, versatile protease regulatable CAR. FKBP DD, FKBP destabilizing domain.

The fluorescein isothiocyanate (FITC)/folic acid switch is one of the "on" switches, mediating the assembly of a tripartite complex by acting as a pseudo-immune synapse between the tumor cells and the CAR-T cells 93. The tripartite complex consists of CAR with anti-FITC scFv, FITC-tagged antibodies, and tumor antigens. The FITC-tagged antibodies recognize tumor antigens and bridge the tumor cells with CAR-T cells. Thus, FITC can redirect CAR-T cells to diverse epitopes of cancer cells, enabling the universal CAR-T and reducing engineering cost (Fig. 3A). Furthermore, the abundance of existing anti-FITC antibody studies and the high affinity of FITC for these antibodies produce a non-negligible advantage for switch design 97. The chemically programmed antibody fragment (cp-Fab)/CAR-T switch was later proposed to improve the original FITC switch and was expected to improve the efficacy of CAR-T in non-small cell lung cancer 98. The design of cp-Fab/CAR-T is characterized by two chemically programmed Fabs, which are conjugated with GCN4 peptide and folate. The cp-Fab bridged anti-GCN4 CAR-T cells with cells highly expressing folate receptors, enabling specific eradication of folate receptor-expressing tumor cells, such as ovarian cancer (Fig. 3A) 99.

Besides bridging CAR-T cells with tumor cells, “on” molecular switches controlling the formation of CAR on the T cell membrane have also been developed. A critical example is the rapalog switch. Rapalog has anticancer properties by binding to the FK506-binding protein 12 (FKBP12) to form a complex, which then binds to the FKBP12-rapamycin binding domain (FRB) on the mammalian target of rapamycin (mTOR) and inhibits mTOR activity. Based on this, a study designed a novel CAR construct with split extracellular scFv and intracellular stimulatory domains. The CAR structure is completed by the addition of the drug rapalog, connecting the FKBP12 and FRB, which are integrated into the intracellular parts of CAR (Fig. 3A) 100. However, the in vivo results of rapalog were not ideal due to the short half-life. For improvement, another study designed an “on” switch with rapamycin, the natural form of rapalog. This study placed the FKBP12-rapamycin-FRB tripartite complex in the extracellular segment of the CAR (Fig. 3A). The results in leukemia tumor cell lines and xenograft models were comparable to second-generation CAR-T cells, indicating the promise for further clinical investigations 101.

The “off” switches control the lysis and degradation of CARs. They can be divided into two types: protein hydrolysis targeted chimera (PROTAC)/versatile protease regulatable (VIPER) systems 102. A study utilizing PROTAC showed promising efficacy in controlling the degradation of CARs, which were constructed with an extra bromodomain (BD) 103. The addition of BD did not change the original functions of CAR-T but it could be recognized by PROTAC compounds, such as ARV771 or ARV825. The compounds ARV771 and ARV825 have been implicated in linking BD-contained proteins to E3 ligases, resulting in protein degradation (Fig. 3B) 104. When the PROTAC compounds were removed, the CAR expression was recovered, indicating the reversibility of this “off” switch. However, this strategy has only been studied in cultured tumor cell lines, with limited results in animal models. Similar to the PROTAC, the VIPER CAR controls the “off” of CAR presentation through the structural domain of a viral protease and its inhibitor, such as the hepatitis C virus NS3 protease (HCV-NS3) and its inhibitor asunaprevir (Fig. 3B) 105,106. The presence of CAR is reversibly controlled by the asunaprevir in a dose-dependent manner in vitro and in vivo. Beyond PROTAC and VIPER systems, other small molecules have also been designed for “off” switches, such as the shield-1. The shield-1 functions by binding to the FKBP destabilizing domain (FKBP DD) (Fig. 3B). The FKBP DD is capable of inducing rapid degradation of the CAR and this process can be inhibited by the shield-1, achieving fine control of CAR presence. The transient rest induced by the removal of shield-1 prevented or reversed the CAR-T exhaustion, enhancing killing persistence in xenograft models 92.

It is worth noting that although these molecular switches have generated exciting results in the precise regulation of CAR-T functions, the related studies were mainly restricted to preclinical models. The clinical applications are significantly hampered by the pharmacokinetics and safety of used small molecules.

#### Micro-environmental Sensing Switch

**Figure 4:**
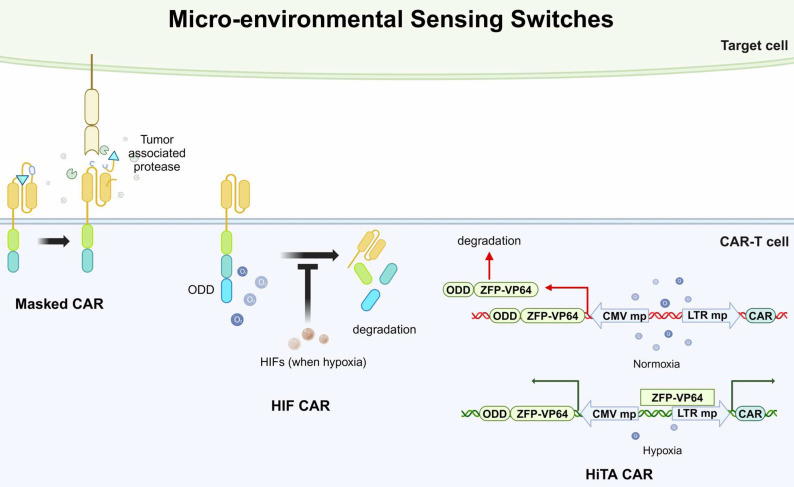
Overview of micro-environmental sensing switches applied in CAR-based killing systems. ODD, oxygen-dependent degradation domain. HIF, hypoxia-inducible factors. HiTA, hypoxia-inducible transcription amplification. ODD, oxygen-dependent degradation domain. ZFP-VP64, zinc-finger protein-VP64. CMV mp, cytomegalovirus minimal promoter. LTR mp, long terminal repeated minimal promoter.

Besides controlling CAR presentation by supplementing small molecules, the CAR can also be specifically activated by sensing factors in the TME, termed the micro-environmental sensing switch. More specifically, strategies of micro-environmental sensing switches aim to exclusively activate CAR-T cells at tumor sites, preventing off-target effects on normal cells. Currently, there are two main approaches to this design: masked CAR and oxygen-sensing CAR 107,108. The masked CARs are switched on and off by an inhibitory peptide. The inhibitory peptide is tethered with the extracellular scFv of CAR and reversibly masks the scFv-mediated tumor recognition. This inhibitory peptide can be degraded by tumor-associated proteases in the TME. Therefore, the CAR-T cells are kept in a dormant state until reach the TME (Fig. 4) 107. Moreover, considering the hypoxic state in the TME, oxygen-sensitive switches have also been considered for CAR-T design. The researchers fused an oxygen-dependent degradation domain (ODD) into the CAR scaffold, which could mediate the degradation of CAR when normoxia. Hypoxia-inducible factors (HIFs) are transcriptional factors induced by a shortage of oxygen supply, which is common in tumor microenvironment. In their design, the HIFs could interact with the ODD and prevent the degradation of CAR (Fig. 4) 108. However, this design was only partially successful in enhancing safety but accompanied by a reduction in cytotoxic efficacy.

Micro-environment sensing switches can also influence CAR expression by regulating transcription. For example, a study utilized the hypoxia state in the TME to regulate the activity of a synthesized transcription factor, zinc finger protein-VP64 (ZFP-VP64). ZFPs are widely applied in biomedical research for their ability to bind to specific DNA sequences and VP64 is a derived form of herpes simplex virus (HSV) proteins, known for its strong transcriptional activation properties. In this study, the researchers fused an ODD sequence with the ZFP-VP64 sequence, therefore the ZFP-VP64 could only stably express and bind to a target sequence when hypoxia. The CAR expression was induced when the ZFP-VP64 bound to the upstream minimal promoter (Fig. 4) 94. This system was termed a hypoxia-inducible transcription amplification system (HiTA-CAR-T). Promisingly, the results exhibited potent antitumor activities while did not induce any systemic toxicity in mice models.

#### Physical Switch

Other than sensing the local environment, strategies that activate CAR expression and presentation by sensing artificially applied physical conditions have also been developed. More specifically, physical switch refers to specialized CAR designs that can only be activated when applying physical signals like irradiation and heat. The advantages of these physical signals are they can activate CAR-T at specific regions and have little risk of off-target effects. Moreover, a physical switch is a promising solution to attack solid tumors, as the physical signals can easily penetrate solid tumor barriers.

**Figure 5:**
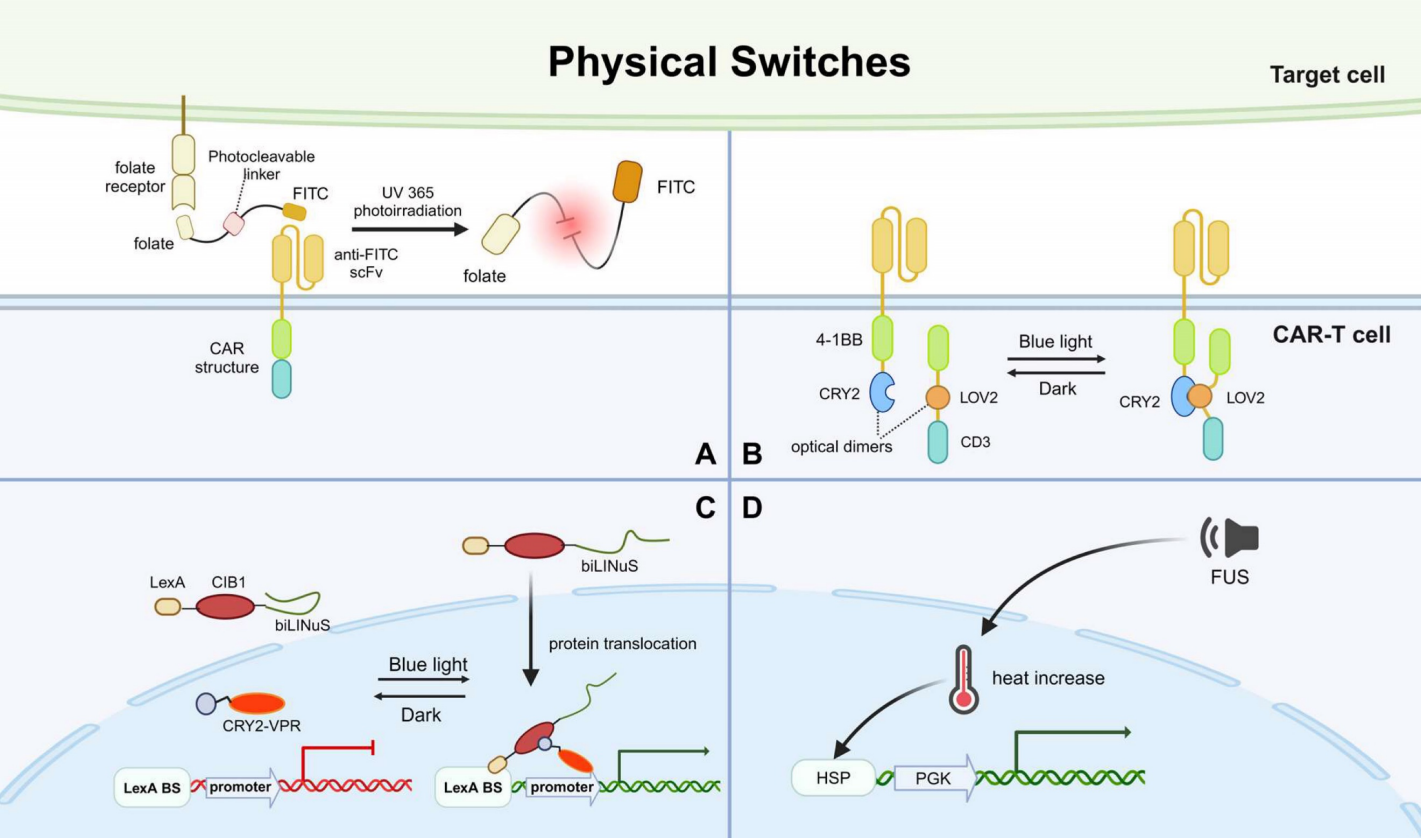
Overview of physical switches applied in CAR-based killing systems. A. CAR targeting effect is mediated by a photocleavable linker, which can self-cleave and abrogate CAR-based killing when UV365 irradiation is applied. B. LiCAR was designed by cleaving the functional domains of CAR intracellularly and ligating a light-responsive module into each half of the construct. C. A protein expression controlling system based on CRY2-CIB1 and LOV2, which can simultaneously mediate protein nuclear translocation upon blue light irradiation. D. An acoustic-controlled CAR-T system based on heat increase generated by FUS. FITC, fluorescein isothiocyanate. UV365, ultraviolet at 365nm. biLINus, bipartite light-inducible nuclear localization signal. CRY2, cryptochrome 2. VPR, viral protein regulatory. LexA BS, LexA binding sequence. FUS, focused ultrasound. HSP, heat shock protein promoter. PGK, mouse phosphoglycerate kinase 1 promoter.

Recently, a photoswitchable approach for regulating CAR-T cells at will was reported. It employs a mediator carrying dual folate and FITC moieties tethered by an ortho-nitrobenzyl ester photocleavable linker 95. When applying UV365 photoirradiation, the photocleavable linker is cleaved and thus, the CAR-T cells are silenced (Fig. 5A). The results showed that this mediator had promising efficacy of bridging CAR-T cells and target cells to induce cytotoxicity in vitro and in vivo. The mediator can split to rapidly terminate the toxicity of CAR-T cells or be re-introduced to re-activate the effects of CAR-T cells. Therefore, a cyclic and precise regulation of CAR-T is achievable.

Another study developed a system based on CRY2-CIB1 and LOV2 that uses blue light to simultaneously control protein nuclear translocation and target protein expression. This system was composed of two protein components, LexA-CIB1-biLINuS (LCB) in the cytoplasm and CRY2-VPR (CV) in the nucleus. Upon blue light irradiation, the helix structure of the LOV2 domain, a part of biLINuS, is unfolded and protein translocation into the nucleus is initiated. After the translocation, LCB binds with CV to form a dimer through CIB1-CRY2 interaction. The LCB-CV dimer is capable of activating the promoter of the target gene and thus, inducing CAR expression (Fig. 5C). The in vitro and in vivo results showed that this system had good efficacy in controlling CAR expression and CAR-T cell cytotoxicity via blue light irradiation. This study further demonstrated that the activation of CAR-T cells at the designated site in mice could be achieved by limiting the area irradiated with blue light 109. However, due to the limited penetration of blue light, this method can only be used to treat solid tumors on the surface of the skin.

Subsequent modulation of CAR-T by optical nanogenetics appears to have solved this problem. Nanophotonics technology is a combination of nanophotonics and optogenetics technology, using nanomaterials as in situ photoconverters to convert long-wavelength excitation light into stimuli that can activate specific ion channels. A novel light-switchable CAR (LiCAR) was designed by cleaving the functional domains of the conventional CAR intracellularly and mounting a light-responsive module into the halves of the separate CARs. Experimentally, it was shown that LiCAR based on CRY2 and LOV2 was the ideal combination (Fig. 5B). Thus, LiCAR-T cells presented light-dependent activation in vitro, indicating that applying light to fine-tune the degree of activation of CAR-T cells is feasible 110. To demonstrate the feasibility in vivo, LiCAR-T cells and surgically removable upconversion nanoplates (UCNPs) that have promoted near-infrared (NIR) to blue upconversion luminescence were further assembled. The UCNPs perform as miniature photosensors that drive LiCAR-T cells to be stimulated by deep tissue-penetrating NIR light in vivo, which can induce activation. This NIR light-tunable nanophotogenetic platform controls CAR-T cell-mediated cytotoxicity spatiotemporally against solid tumors and hematologic malignancies with customized dosing and duration, thus alleviating the side effects caused by current immunotherapies 110.

To address the issue of light penetration, an acoustic-controlled CAR-T was developed. The researchers constructed a vector containing a heat shock protein promoter (Hsp) and a mCherry driven by constitutive mouse phosphoglycerate kinase 1 promoter (PGK), which can induce CAR expression. Hsp can be activated during local heat shock and sequentially promotes the expression of CAR. When the team used focused ultrasound (FUS), the FUS-converted thermal energy raised local heat and activated Hsp, leading to CAR expression (Fig. 5D). Without any exogenous cofactors, the FUS-activated CAR-T cells at specific times and deep tissue sites to precisely inhibit solid tumor growth in vivo 111. A synthetic genetic switch based on photothermal control has also been described and used to demonstrate local photothermal control of engineered T cell activity 112.

#### Suicide Switch

Despite the efficient cytotoxicity of CAR-T cells, CAR-T-induced targeted cell pyroptosis is still a problem as it is suspected with CRS induction 113. Moreover, in patients receiving anti-CD19 CAR-T therapy, the CAR-T cells were found to kill normal CD19+ B cells after depleting malignant B cells 114. The suicide switch induces CAR-T cell apoptosis upon activation by designed signals. The incorporation of suicide genes in CAR-T cells is an effective means to restrict serious adverse events driven by CAR-T over-killing.

**Figure 6:**
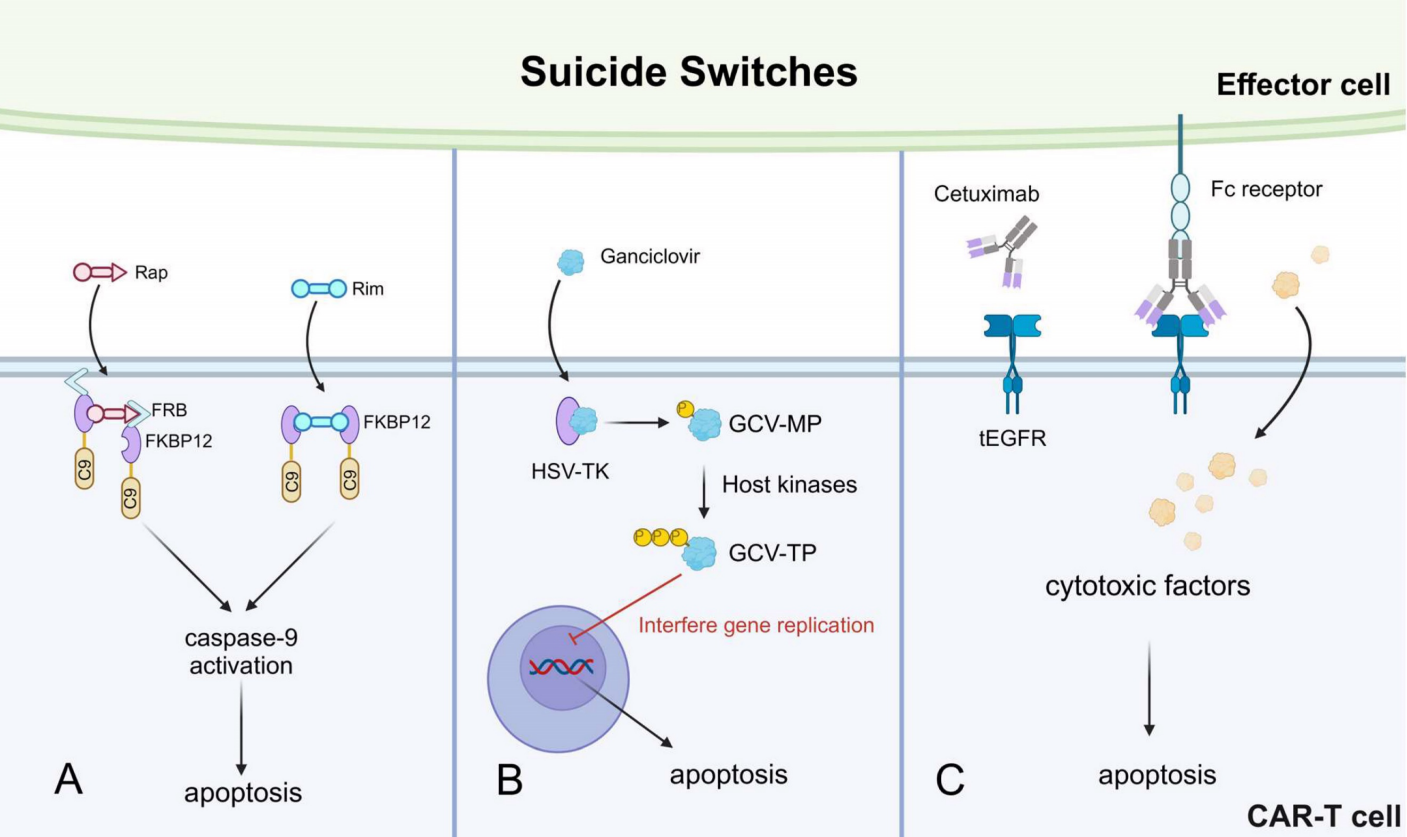
Overview of suicide switches applied in CAR-based killing systems. A. A safety switch designed to activate caspase-9 and apoptosis, mediated by Rap and Rim. B. A suicide switch utilizing herpes simplex virus in T cells. C. A truncated form of epidermal growth factor receptor (tEGFR) acts as an epitope handle that can be used to target T cells with a mAb such as cetuximab. Rim, rimiducide. C9, caspase9. Rap, rapamycin. HSV-TK, thymidine kinase in herpes simplex virus. GCV-MP, ganciclovir monophosphate. GCV-TP, ganciclovir triphosphate.

An iCaspase 9 suicide gene system has been described in detail previously and applied in a phase I clinical trial, where the iCaspase 9 safety switch was combined with CAR-T and showed promising results 115,116. Based on this, a study developed a rapamycin-induced, caspase9-based suicide switch (iRC9). As discussed above, the rapamycin can bind with FKBP12 and FRB to form a tripartite complex. The researchers utilized this feature to construct an FRB-FKBP12-caspase9 complex (Fig. 6A). The addition of rapamycin led to the dimerization of this capsase9-containing complex, resulting in apoptosis 117. Similarly, the same group also developed another caspase9-based switch by rimiducid, which facilitated the dimerization of the synthesized FKBP12-caspase9 complex, also leading to apoptosis (Fig. 6A).

Other suicide switches have also been described. For instance, the HSV thymidine kinase (HSV-TK)-mediated suicide switch 118. Ganciclovir (GCV) is a nucleoside analog that can be phosphorylated by the HSV-TK into the GCV-monophosphate (MP) form, which is then further transformed into GCV-triphosphate (TP) by host kinases. The GCV-TP incorporates into the nascent DNA chain during gene replication and interferes with the replication process, leading to CAR-T apoptosis (Fig. 6B) 119. However, this suicide switch is significantly limited by immunogenicity. Multiple immunogenic epitopes were identified in T cells expressing HSV-TK, leading to CD4+ T cell and CD8+ T cell-mediated anti-CAR-T responses 96. Another synthetic suicide switch was described as a truncated form of epidermal growth factor receptor (tEGFR) expressed on the surface of CAR-T cells. The tEGFR can be recognized by the monoclonal antibody cetuximab, which binds to tEGFR and recruits effector cells to kill the CAR-T cells (Fig. 6C) 114. This approach has successfully prevented CAR-T-mediated depletion of normal B cells in xenograft mice models, meanwhile without tumor relapse. Therefore, this suicide switch is promising for controlling persisting CAR-T cytotoxicity-related side effects in clinical patients.

Different switches discussed in this section should be applied to different conditions. For example, microenvironment sensing and physical switches are more suitable for solid tumor treatment, as discussed above. To reversibly control CAR-T “on” and “off”, molecular switches are more suitable as the switching can be easily manipulated by supplementing and depleting drugs. To avoid over-killing of CAR-T in patients who already exhibit tumor remission, suicide switches can mediate the irreversible termination of CAR-T treatment. However, as most of these novel designs are still in preclinical models, many questions remain to be solved for further clinical considerations. The pharmacokinetics of small molecules, the safety of irradiation, and the suitable clinical window for turning off CAR-T cells are all important questions that warrant further research.

### Logic Gate Circuit for Refining Antigen Recognition

Off-target effects are one of the main side effects of cellular immunotherapies. Off-target effects can occur when CAR-T cells mistakenly injure normal tissue cells that also express the target antigens, leading to extra tissue damage or immunodeficiency. Screening for suitable antigens that are exclusively expressed by tumor cells is difficult, and most antigens utilized by current CAR-T therapies have high expressions in tumor cells while low expressions in normal cells. For example, the anti-CD19 CAR-T cells have shown great cytotoxicity in depleting leukemic B cells, while some brain cells also express the CD19 protein and are recognized by the CAR-T cells, leading to neurotoxicity 120. To address this problem, diverse logic gate circuits have been developed to increase the specificity of CAR-T cells against tumor cells.

#### And

**Figure 7:**
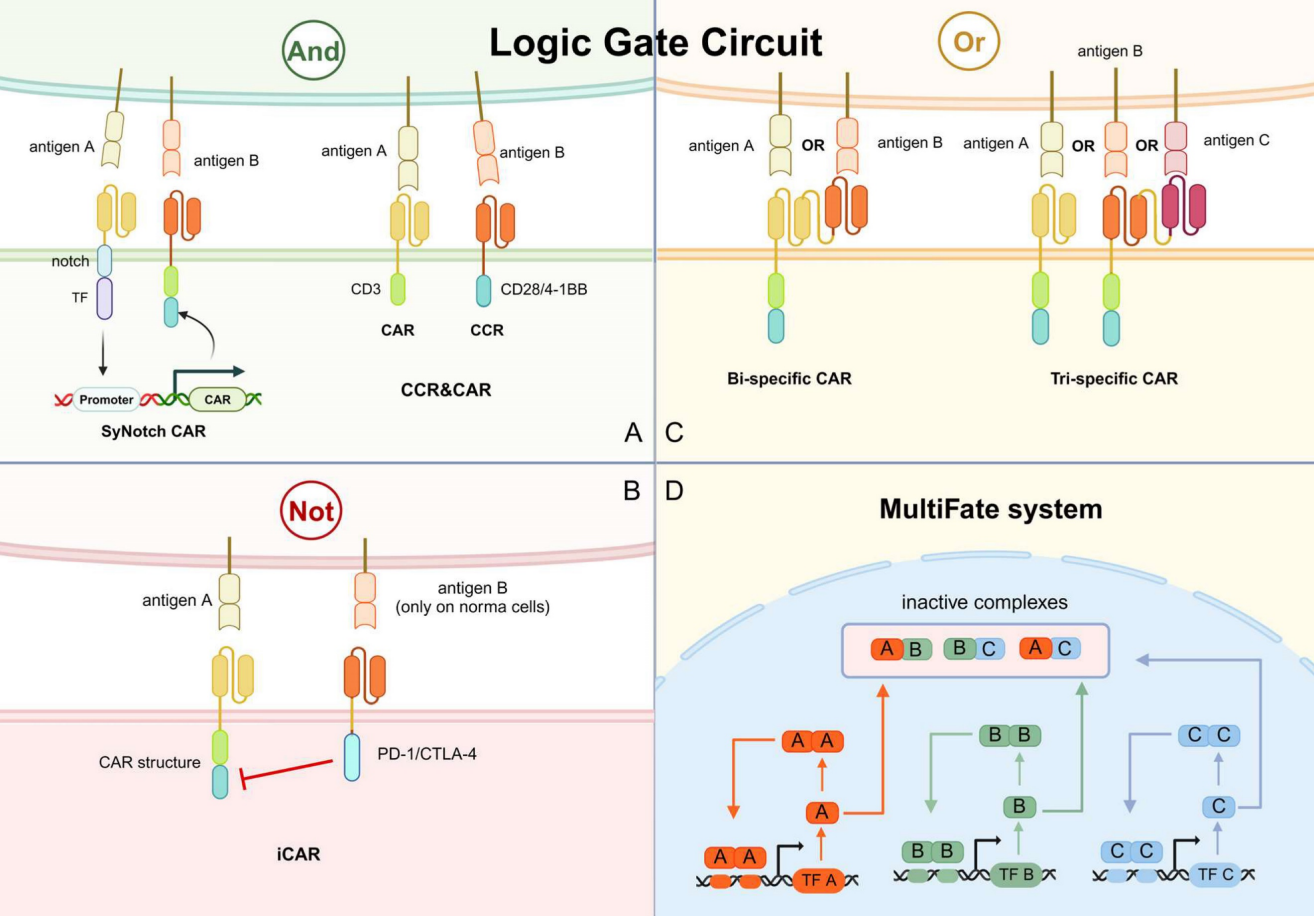
Overview of logic gate circuit applied in CAR-based killing systems. A. The SyNotch CAR and CCR&CAR for “and” logic gate circuits. B. The iCAR for “not” logic gate circuits. C. The bi-specific and tri-specific CAR for “or” logic gate circuits. D. The MultiFate system for switching T cell states. TF, transcription factor. CCR, co-expression of chimeric stimulated receptor.

The design of “and” logic gate circuits emphasizes the idea that CAR-T cells should be exclusively activated towards tumor cells by sensing dual or multiple antigen signals on the tumor cell surface. The problem that CAR-T cells recognize normal tissues is avoided by the sufficient sensation of multi-antigen (Fig. 7A).

Notch-based assembled receptor (SynNotch) is one of the main designs of “and” logic gate circuits. The SynNotch receptor contains scFv against one tumor antigen and a transcription factor domain, which is cleaved and released when the receptor recognizes the tumor antigen. The release transcription factor stimulates the expression of CAR, which targets another tumor antigen (Fig. 7A) 11. Since individual SynNotch pathways are designed to activate different signaling intermediates, multiple SynNotch receptors can be used in the same cell to achieve multiple-targeting of CAR-T cells 121. For instance, the SynNotch-based GD2-B7H3 CAR-T cells regulate metastatic xenograft mouse models and neuroblastoma growth in vitro with high metabolic adaptability, specificity, and potency 122. However, this system has several flaws that hinder its application in clinical practice. Due to its non-human component, the SynNotch receptor has a high potential to trigger immunogenicity in the host. In addition, it lacks clear design rules to construct well-expressed receptors with modifiable activity. The large size of its receptor and transcriptional line also limits its clinical application.

Another option is dual targeting by co-expression of a first-generation CAR and a chimeric costimulatory receptor (CCR). The CCR contains scFv with 4-1BB or CD28 (Fig. 7A). CD3ζ activation stimulates the first signal of T cell activation, while 4-1BB or CD28 activation provides the T cell activation second signal. The combination of CAR and CCR constitutes a multiple targeting and co-stimulation strategy to improve the clinical outcome of CAR-T cells by improving cytotoxic efficacy and persistence while limiting the off-target effects 123.

#### Not

Another way to avoid mishaps is to design “not” logic gate lines. An extra receptor, termed inhibitory CAR (iCAR), recognizing antigens present in normal cells and inducing inhibitory signals is designed in “not” logic gate circuits. The PD-1/CTLA-4-based iCAR strategy was previously described by tuning iCAR targets to normal cell antigens (Fig. 7B). The iCAR-expressing CAR-T cells can distinguish between normal and target cells and function transiently and reversibly 124. For example, the toxicity of anti-CD19 CAR-T against normal B cells is discussed above. Malignant B cells have been reported to downregulate surface HLA-C1 expression, therefore HLA-C1 can be a candidate for distinguishing normal B cells from malignant B cells. A study has developed PD-1 iCAR against HLA-C1 and results showed comparable cytotoxicity against mice leukemia cells while reduced normal HLA-C1^+^ B cell depletion 125.

#### Or

Tumor relapse is a significant problem after CAR-T therapy, and the relapsed tumor cells often exhibit antigen loss or antigen heterogeneity. In a classic follow-up report, up to 50% of patients with acute lymphoblastic leukemia treated with anti-CD19-CAR-T cells relapsed within the first year, and a significant proportion of them exhibited CD19 antigen loss 126. The “or” logic gate circuit allows CAR-T cells to recognize multiple antigens but signals from each antigen are sufficient to activate the CAR-T cells. This design is promising to solve the problem of antigen loss and antigen heterogeneity.

Bi-specific CAR containing two linked scFvs have been designed for this “or” logic gate circuit (Fig. 7C). CAR-T cells in tandem with anti-CD19 scFv and anti-CD20 scFv can effectively prevent antigen evasion by malignant B cells 127. Activation of either scFv can lead to CAR-T activation and cytotoxicity. CAR-T cells with anti-BCMA and anti-CD38 scFvs have also been shown to robustly control multiple myeloma 128. This design was subsequently expanded to tri-specific CAR, targeting CD19, CD20, and CD22 to effectively eliminate B-cell tumors with heterogeneous antigens in preclinical models (Fig. 7C) 129. Notably, the application of the "or" logic gate in CAR-T has also been effective in alleviating solid tumors, which are highly antigenic heterogeneous. For example, CAR-T targeting both GD2 and B7-H3 achieved rapid and sustained anti-tumor effects in mice implanted with human neuroblastoma tissue 130. Notably, the large size of the “or” receptors might be a significant problem limiting their further application.

#### MultiFate system

T cells in tumors usually exhibit three different states: resting, activated, and depleted. The ultimate goal of synthetic immunotherapy is to create a CAR-based killing system with a synthetically regulated gene network, achieving the switching between different states. The feasibility has been demonstrated in previous studies, and this strategy was denoted as the MultiFate system 131. In the MultiFate system, transcription factors have a shared common dimeric structural domain that allows them to form homodimers and heterodimers competitively. The promoters of every transcription factor gene contain binding sites only strongly bounded by their homodimers, making homodimer-dependent self-activation possible (Fig. 7D). On the contrary, heterodimers cannot bind efficiently to any promoter in this design. Thus, the heterodimers act to mutually repress the activity of the two constituent transcription factors. The genes encoded by each transcription factor are responsible for different CAR-T states and by simply supplementing corresponding transcription factors, the states can be changed 131. This system thoroughly exploits the value of immune cells and artificially manipulates the functions of CAR beyond just “activation” or “death”. However, as an innovative strategy with complex genetic designs, the feasibility has not been verified in cell or animal models.

### Other Synthetic Molecules for Enhancing Immunotherapies

#### Cytokines that Enhance Immune Responses

As mentioned above, antigen downregulation or antigen loss can lead to tumor relapse, which is a significant obstacle to CAR-T therapy 132. Antigen-negative cancer cells that are not recognized by CAR-T cells can be eliminated by NK cells or CIK cells 34. To address this problem faced by CAR-T therapies, the TRUCK strategy was developed to recruit and activate innate effector cells into tumor lesions by CAR-T-derived cytokines. Diverse cytokines have been applied in cellular immunotherapies, as summarized in Table 2. The CAR-T cells in the TRUCK strategy are specially engineered with a constitutive expression cassette for specific cytokines, IL-12 for instance 133,134. In TRUCK, IL-12 expression in CAR-T cells is regulated by the nuclear factor of activated T cells (NFAT) and secreted only when CAR-T cells are activated. Thus, IL-12 specifically accumulates in tumor tissues. IL-12 enhances T cell activation, modulates the vascular tumor microenvironment, and recruits various immune cells to fight against cancer cells that are not recognized by CAR-T cells. The restricted secretion of IL-12 in CAR-targeting tissues also prevents the side effects generated by the over-release of IL-12 in normal tissues 134. Synthetic cytokine (IL-2) circuits designed based on the TRUCK strategy and SynNotch (the “and” logic gate circuit) have also been applied in CAR-T research. This targeted IL-2 delivery circuit offers a promising approach to overcome tumor suppression locally and minimize systemic IL-2 toxicity at the same time. The orthogonality of SynNotch also has more synthetic potential compared to TRUCK. The investigators also explored the impact of autocrine and paracrine cytokines (both based on T cells) on efficacy and found that the autocrine cytokine system was more effective, possibly because the autocrine system has access to the greatest number of cytokines to improve its activity 135.

Besides IL-12 and IL-2, other cytokines with immune stimulatory effects have also been applied in CAR-T research 136. IL-18, primarily released by macrophages and DCs, activates Th1 and NK cells to release IFN-γ, enhancing CAR-T cell function and activating the endogenous immune system. With TRUCK, IL-18 can be secreted by intra-tumor CAR-T cells and generate similar efficiency of tumor eradication and mice survival compared with IL-12 137. IL-15 stimulates CD8+ T cells and NK cells, enhancing the anti-tumor activity of adoptively transferred T cells. CAR-T cells engineered to secrete IL-15 exhibit augmented tumor cytotoxicity, increased T cell expansion and persistence, and greater protection against tumor recurrence. Compared to constitutive secretion of IL-15 by CAR-T cells, inducible or tethered IL-15 may be better to avoid high systemic levels of it, which could result in systemic toxicity 138-140. IL-7 promotes the proliferation and function of CAR-T cells without enhancing Treg activity. Enhancing CAR T cell function in solid tumors and partly preventing suppression by immunosuppressive cytokines in the TME also have promising applications for integration into CARs 141-143.

#### Neutralizing Antibodies

In addition to releasing cytokines, engineering T cells to secrete neutralizing antibodies can also help improve their therapeutic effectiveness. First, CAR-T cells are designed to secrete PD-1 scFv to enhance the efficacy of CAR-T cell therapy against poorly responsive tumors, also known as the combined application of cytotoxic cells and immune checkpoint inhibitors 135. These scFv-secreting CAR-T cells function in both paracrine and autocrine manners to enhance the anti-tumor activity of CAR-T cells and endogenous T cells. It is worth noting that the efficacy of this strategy is better and safer compared to that of CAR-T cells or checkpoint inhibitors alone, as the secreted PD-1 scFvs still localize to the tumor, avoiding systemic checkpoint inhibitor-associated toxicity. On the other hand, neutralizing key cytokines that trigger side effects (especially CRS) through antibodies can greatly improve the safety of immunotherapy. The central cytokines involved in CRS are IL-1, IL-6, and GM-CSF. In CAR-T therapy, CRS-related IL-1 and IL-6 are mainly derived from monocytes 144. However, the main source of GM-CSF is CAR-T cells. A previous study demonstrated a CAR-T cell that constitutively produces IL-1 receptor antagonists (IL-1RA) that protected mice from CRS-related mortality without compromising anti-tumor efficacy 145. The secretion of anti-IL-6 scFv and IL-1RA by CAR-T can self-neutralize IL-6 storms and maintain low levels of IL-1β, minimizing IL-6-and IL-1-related cytokine toxicity and neurotoxicity 146. A subsequent novel safe T cells (SAFET) platform built on this basis by knocking out the GM-CSF gene further reduces cytokine toxicity 147. A recent study showed that CRISPR/Cas9-mediated GM-CSF knockdown in CAR-T cells directly improved early activation of CAR-T cells and enhanced anti-tumor activity in preclinical models 148. Therefore, further modulation of cytokine release without compromising the active function of CAR-T cells can effectively avoid the risk of side effects such as CRS and thus enhance clinical outcomes. Compared with the switch and logic gate modulation discussed above, the self-neutralizing cytokine strategy is less sensitive but has more potential for clinical application because it does not involve non-human-derived proteins.

#### Dominant-negative Receptor (DNR)

Transmigrated T cells are inherently susceptible to immunosuppression, therefore blocking the immune checkpoint pathways like PD-1 by genetic engineering allows for the long-term persistence of modified cells with sustainable cytotoxicity and proliferation in vivo. The success of antibody-mediated checkpoint blockade requires a relatively high mutational burden and the presence of infiltrating T cells 149,150. Inspired by this, DNRs to inhibit PD-1 signaling have been proposed. PD-1 DNR is a modified form of endogenous PD-1 receptor and the DNR lacks the intracellular signaling domains. Therefore, the PD-1 DNR can compete against the PD-1 receptor for extracellular PD-1 but does not generate any cellular events after binding 151. When co-transduced with second-generation CARs, the PD-1 DNR prevented the PD-1-mediated T cell suppression and enhanced CAR-T cell functional persistence. The success in solid tumor models also demonstrated the feasibility of providing both co-stimulation and checkpoint blockade to counteract the immunosuppressive TME in solid tumors.

Besides immune checkpoints, many cancers also produce an immunosuppressive environment primarily with TGF-β 152. TGF-β DNR has been long developed to reduce the inhibitory effects of tumor-derived TGF-β on cytotoxic T lymphocytes 153. In 2010, a clinical trial (NCT01140373) using prostate-specific membrane antigen (PSMA)-targeted second-generation CAR-T cells for prostate cancer reported limited persistence and no significant responses. Later, a study blocked the TGF-β signaling via TGF-β DNR and the results exhibited enhanced tumor infiltration, proliferation, persistence, and efficacy of anti-PSMA CAR-T therapy in mice models 154. A new phase I clinical trial using anti-PSMA TGF-β-DNR CAR-T cells in prostate cancer was reported with the feasibility and safety of the clinical application of TGF-β-DNR CAR-T cells 155. Further research is required to assess the long-term efficacy and safety of DNR CAR-T cell therapy in larger patient populations.

#### Drug-conjugating Enzyme

Anti-tumor small-molecule drugs have been extensively studied, such as carboxypeptidase G2 (CPG2), and Enterobacter cloacae β-lactamase (β-Lac) 156,157. Generating small molecule drugs locally at the tumor site would diminish the toxicity related to systemic drug delivery as well. Various methods to deliver antigen-targeted drugs have been previously developed. The synthetic enzyme-armed killer (SEAKER) CAR-T was recently proposed and validated based on the desire to apply CAR-T cell immune function in synergy with drugs 158. The system works by releasing recombinant enzymes, to activate the activity of the pro-drug. Even in the case of CAR-T depletion, the recombinant enzyme is still produced to promote drug killing of antigen-negative tumor cells. Compared to bacterial delivery vectors, ADEPT utilizes the targeting properties of CAR-T cells, facilitating precise drug delivery. It has been observed that when combining the killing effect of a drug with CAR-T, the population of CAR-T cells is reduced, which in turn reduces the preclinical drug preparation cycle and risk of CRS 158. However, as the recombinant enzymes are constitutively incorporated with CAR-T cells, there are still possibilities of off-target toxicity. Moreover, the immunogenicity of the recombinant enzyme is also a significant problem. Adverse results could also be generated by the consistent release of recombinant enzymes, even if tumor cells have already been eradicated.

## Synthetic Delivery Systems for Enhancing Cellular Immunotherapies

### Hydrogels for CAR-T Cell Delivery

In addition to the synthetic cells and molecules mentioned above, there are also other novel synthetic biomaterials being investigated to enhance CAR-T efficacy. The feasibility of using synthetic biomaterials with immunostimulatory factors to deliver transferred T cells has been validated in previous studies, exhibiting promoted T cell proliferation and tumor infiltration 159. However, such strategies often involve the fabrication of complex biomaterials or surgical implantation, which are technology-demanding and costly. By contrast, hydrogels have superior biocompatibility, tunable physicochemical and mechanical properties, and environment-sensing abilities, making them suitable for CAR-T cell delivery 12,160-162. More specifically, hydrogels in CAR-T therapies can immobilize active T cells by providing appropriate physical support, effectively enhancing CAR-T efficacy 12. Meanwhile, hydrogels can also function as immunoadjuvants to improve the immunogenicity of tumor antigens and promote T cell activation 163. Hydrogels also provide a three-dimensional growth environment for the development and delivery of CAR-T cells, protecting the CAR-T cells from the immunosuppressive factors. The tunable properties of hydrogels also make it easy to manipulate CAR-T cell distribution, activity, and targeted release.

Various types of hydrogels have been used to deliver therapeutic immune cells and cytokines. Thermosensitive hydrogels are the most commonly used type, but they have certain limitations, such as an unregulated curing process after injection and low-temperature encapsulation conditions that can affect cell viability 164. Injectable and photocurable gelatin methacrylate-based hydrogels have been proposed as a novel repository for CAR-T cells 12. Another self-assembled and injectable biomaterial platform based on polymeric nanoparticle (PNP) hydrogels for CAR-T cell and cytokine delivery has also been developed 165. In response to hypoxia in the TME, an injectable hydrogel-encapsulated porous immune microchip system ameliorates hypoxia in TME by carrying HEMOXCell (a marine extracellular hemoglobin with excellent oxygen storage capacity) and IL-15 166. The application of hydrogels enables the in vivo expansion of more tumor-responsive CAR-T cells by providing niches and cytokines, thus significantly decreasing the number of therapeutic cells required during the manufacturing phase and reducing costs.

All the synthetic molecules discussed in previous sections are tuned to precisely control CAR-T expression and activation, but they all involve non-human proteins or large gene constructs. One prominent advantage of using hydrogels in CAR-T therapies is that hydrogels can control the rate and location of CAR-T cell release, preventing the over-activation and off-target effects of CAR-T cells 166,167. Based on many inherently desirable properties, bio-responsive multifunctional hydrogels can perform complex biological functions, such as sensing the acidic and hyaluronidase-rich environment of tumors and releasing anti-tumor chemotherapeutic agents. It therefore has the potential to switch off the effect of CAR-T cells by sensing the tumor environment, thus achieving a function similar to that of a suicide switch. Moreover, except for cells, hydrogels can also be loaded with cytokines such as the IL-12 discussed above. Functional cytokines can be simply mixed with CAR-T cells in the hydrogels and released at the desired sites, which is more convenient than the transgenic methods. The site-specific releasing property can also prevent the over-release of functional cytokines, avoiding CRS without harming the cytotoxicity 12.

However, hydrogels in CAR-T therapies are a relatively new field and the amount of research is relatively limited, therefore it is still unclear whether hydrogels are a more suitable option for clinical applications than transgenic modifications of CAR-T. Further studies and clinical trials are necessary to fully evaluate the potential benefits and drawbacks of each approach.

### In Vivo Delivery for CAR-T Production

Current approaches to delivering CAR genes to immune cells include the employment of viral vectors, transposons, or electroporation. The classical CAR-T preparation route includes extraction of T cells, activation, modification, amplification, and transfusion back. This results in a very high cost and long preparation time for CAR-T therapies. To solve the problems, allogeneic universal CAR-T, CAR-NK, and other therapies are developing rapidly. Also, the production of CAR-T cells appears to be more desirable in vivo than general-purpose CAR-T because they do not cause GvHD and host-versus-graft response (HvGR). In vivo production of CAR-T has begun to evolve from the generation of CAR-T cells through polymer nanoparticles (PNP) completing in situ programming to the transient production of CAR-T cells through lipid nanoparticle (LNP) delivery of mRNA 13. Targeting is central to accomplishing in vivo delivery of CARs. Four modalities regarding in vivo production of CAR-T: LV, AAV, mRNA-nanocarriers, and mRNA-LNP, have all been validated and discussed in previous reviews 168-172. In addition, a multifunctional alginate scaffold for T cell engineering and release (MASTER) has been shown to rapidly produce and release CAR-T cells in vivo and reduce the time to manufacture CAR-T to 1 day 173. It preserves the traditional CAR-T production process while shortening the overall production cycle. Furthermore, there are also various other in vivo engineering platforms for CAR-T, as summarized in Table 3.

This in vivo delivery for in situ programming idea has been extended to engineered macrophages, which is more applicable in solid tumors. The nanocomplexes of macrophage-targeting nanocarriers and CAR-IFN-γ-encoding plasmid DNA can be injected in vivo to induce CAR-macrophages that are adequate for anti-tumor immunomodulation, CAR-mediated cancer phagocytosis, and inhibition of solid tumor growth. In summary, the off-the-shelf CAR-macrophage therapy is effective against solid tumors and avoids the complicated and expensive ex vivo CAR-cell manufacturing process 174. In situ, editing of "local" macrophages around the postoperative tumor cavity is performed by co-delivery of macrophage-targeted editing nanocarriers (pCAR-NPs) and CD47 antibodies in a "filled" form. The pCAR-NPs and antibodies are delivered by hydrogels manufactured to generate CAR-macrophages around the tumor cavity 175. It is worth noting that the in vivo production of the CAR-cell strategy cannot overcome the immune debilitation of the patient. Therefore, like conventional CAR-T cell therapy, this treatment has certain requirements for the patient's immunity condition.

### Exosomes for In Vivo Production of CAR-T Cells

We also focus on the recently emerging delivery vector, exosomes, although there are no studies on the in vivo production of CAR-T through them currently. As extracellular vesicles secreted by cells, exosomes are intercellular signaling bodies for intercellular communication and pharmacological actions. Due to their naturally abundant presence in the body, low toxicity, and the ability to encapsulate endogenous bioactive molecules, exosomes have great therapeutic potential in cancer therapy. Using engineering techniques, exosomes can acquire aggressive targeting capabilities by attaching targeting units to membrane surfaces or loading them into luminal bodies to accumulate in specific cell types and tissues 176. Recent studies have described the delivery of cytotoxic proteins and activation of the cysteine signaling pathway via neutrophil-derived exosomes (N-Ex) to induce apoptosis in tumor cells 177.

Regarding the use of exosomes in CAR-related immunotherapy, previous studies have confirmed that CAR exosomes from effector CAR-T cells possess powerful anti-tumor effects and low toxicity. High levels of CAR expression were reported to be produced in such exosomes and they carried granzyme B and perforin. Since CAR exosomes do not express PD-1 compared to CAR-T cells, recombinant PD-L1 treatment could not diminish their anti-tumor effects. Furthermore, in CRS animal models, the administration of CAR exosomes was relatively safer compared to CAR-T treatment 178. Another study reported that HEK293T-derived anti-CD19 CAR-exosomes induced elevated cytotoxicity and pro-apoptotic genes in CD19-positive leukemia B cells without inducing cell death in CD19-negative cells 179. This is similar to the mechanism of CAR-MSC-induced target cell death described above. Among the applications of CAR-associated immunotherapies, in vivo production of CAR-T by exosomes does not seem to have been studied. However, the other two possible applications have been reported. Considering that exosomes have shown exciting efficacy in other studies of drug delivery or transient transduction in vivo, we believe that the use of exosomes in immunotherapy could effectively solve the problem of cost or efficacy.

## Discussion

Cellular immunotherapies involving genetic modifications of intrinsic immune cells have revolutionized the treatment of cancers. Diverse novel synthetic strategies have been proposed in recent years to modulate or enhance the function of engineered immune cells, concerning engineered cells, synthetic molecules, and synthetic biomaterials for delivery. CAR-T and TCR-T therapies are the prominent representatives of T cell-based cellular immunotherapies, with each having distinct scenarios for suitable applications. The structural designs of CAR-T and TCR-T have evolved through many generations, with each new design targeting a specific problem, such as the SUPRA CAR for achieving universal CAR-T targeting. A variety of immune cells beyond T cells are being engineered in research for the treatment of different diseases, the most central being CAR-based cytotoxic immune cells, with CAR-NK receiving the most attention and considered to be a promising candidate for the next generation of cellular immunotherapy. In addition, other adjuvant immune cells like DCs, neutrophils, and MSCs have also been engineered for immuno-modulatory potentials 84. Synthetic molecules participating in the fine-tuning control of CAR expression and activation have also received much attention, functioning in the form of precise switches, logic gate circuits, DNRs, etc. The synthetic molecules discussed above nearly all require extra genetic constructs to achieve their functions, which significantly adds to the engineering difficulty and cost. The tailored nove biomaterials such as hydrogels seem to be promising for achieving the purposes of those designed switches and circuits while sparing the difficulty and cost for extra engineering. Further research is warranted to assess the advantages and disadvantages of using hydrogels for CAR-T delivery and functional enhancement, compared to the iterations of CAR structures and synthetic molecules.

The synthetic strategies discussed above nearly all focus on the fine-tuning of antigen recognition and CAR expression. More novel strategies modulating other aspects could be proposed by the advancements in the understanding of immune response mechanisms. For example, the mitochondria-related metabolism in CAR-T cells has been acknowledged to significantly affect CAR-T cell exhaustion, cytotoxicity, and tumor relapse 180. Recently, a study identified an FDA-approved drug, enasidenib, can enhance memory CAR-T cell formation and sustain cytotoxicity in vivo by inhibiting the activity of an endogenous enzyme, which interrupts glucose utilization and increases oxidative stress in CAR-T cells 181. Therefore, it is promising to develop synthetic strategies to target metabolism components to alleviate metabolic stresses and exhaustion of T cells.

The advancement of biotechnologies is also a crucial factor in accelerating the development of cellular immunotherapy. In addition to the technological advancements discussed above, the cooperation of CAR-T cells with organoids has gained much attention recently. The ability of organoids to highly recapitulate the original characteristics of tumors in vitro holds great promise for therapy research, predicting patient response to therapy, and providing personalized medical regimens for patients. T lymphocytes derived from healthy blood donors can be effectively expanded and activated in vitro using organoids. These activated T cells can be employed for in vivo patient treatment, potentially facilitating in vitro assessment of the cytotoxicity of healthy donor-derived T cells on patient-derived tumor-like cells. Organoids and immune cells in vitro co-culture can expand immune cells and enhance immune responses, providing a strong guarantee for subsequent immunotherapy 182.

Immune remodeling is currently a promising strategy for fighting disease, as weakened immunity (also called immunosenescence) indirectly contributes to disease. The primary objective of synthetic immunotherapy is to optimize outcomes across diverse diseases, shortening treatment cycles, lowering cost, and minimizing relapse and side effects. Although many clinical hurdles remain to be overcome, it is indisputable that synthetic biology holds great promise for advancing precise medicine by synthesizing cells and molecules to achieve specialized, efficient, and controlled treatments for complex diseases.

## Figures and Tables

**Table 1:** Comparison between CAR-T and TCR-T

	CAR-T	TCR-T	References
**Target Antigen**	Surface antigens presented on tumor cells	Both intracellular and extracellular antigens presented by MHC class Ⅰ	15
**Binding Affinity**	Fixed affinity determined by the CAR structure	Variable affinity determined by the TCR specificity to the presented MHC	17
**Killing Characteristics**	Relatively quick sequential killing	Slower but longer-lasting killing signal	22
**Advantages**	1. Stronger killing ability. 2. MHC-independent recognition. 3. Relatively easier engineering process. 4. Lower risk of autoimmunity.	1. Higher binding affinity to the target. 2. Longer-lasting killing signal. 3. MHC-dependent recognition might provide a wider range of targets. 4. Better efficacy in solid tumor models.	17
**Disadvantages**	1. Restricted efficacy in solid tumor models. 2. Risks of CRS and neurotoxicity. 3. Limited efficacy in treating tumors with heterogeneous antigen expression.	1. Screening TCR specific for homologous MHC increases the engineering time and cost. 2. Higher risks of off-target toxicity and autoimmunity. 3. Lower killing efficacy.	17,183

**Table 2:** Cytokines functioned in immunotherapies

Cytokine	Source	Function(s)	Application(s)	References
IL-2	CD4+ T cells and CD8+ T cells	Promote proliferation and differentiation of CD8+ T cells into CTLs; stimulate cytotoxicity and cytokine production of CTLs and NK cells.	**High dose IL-2:** Metastatic renal cell carcinoma and metastatic melanoma approved by FDA.	184-186
			Pulse intermediate dose IL-2: Increase cytotoxic activity of NK cells.	
			Pulse intermediate dose IL-2 and rituximab: Enhanced ADCC mediated by NK cells. But no significant clinical benefits in other studies.	
			**IL-2-based immunocytokines:** Preclinical lymphoma studies include IL-19-IL-2 in NHL, HI-Leu 16-IL-2 in lymphoma, and HRS3scFv-IL-12-Fc-IL-12 in HL. Diphtheria toxin-IL-2 fused proteins in Phase II/III trials of CTCL, refractory/relapsed CTCL, and peripheral T cell lymphomas.	
IL-7	Stromal cells and DCs	Stimulate differentiation of multipotent hematopoietic stem cells into lymphoid progenitor cells, and promote CAR-T cell proliferation and function without impacting Tregs.	**IL-7 secretion induced by CAR-T cell engagement:** Amplify CAR-T cells in the presence of TGF-β; partly prevent suppression by immunosuppressive cytokines in the TME.	141,142
IL-12	DCs, monocytes, macrophages	Augment the cytolytic activity of T cells and NK cells; enhance the expression of MHC class I molecules; enhance anti-solid tumor efficacy in the TME.	**Injection of IL-12 after autologous peripheral blood stem cell transplantation:** Time to progression with a median of 32.4 months in 3/8 of the NHL patients and 1/2 of HL patients.	90,187,188
			**IL-12 secreting tumor-targeted CAR-T cells:** Enhance anti-tumor efficacy in vitro and in vivo; induce a bystander effect in host immune cells in vivo, providing immune memory to the cancer antigen. However effective doses for systemic administration of IL-12 were highly toxic.	
			**T cells with inducible IL-12 secretion upon CAR engagement:** Enhance tumor cytotoxicity; induce a bystander anti-tumor response; enable cytotoxicity of various cells within the solid tumor, irrespective of antigen expression.	
IL-15	Macrophages, monocytes, DCs, etc.	Enhance the proliferation and survival of T cells, proliferation and differentiation of NK cells, and differentiation of CTL; Prolong expansion and activation of both NK cells and CD8+ T cells.	**Recombinant human interleukin-15:** First-in-human clinical trial of metastatic cancers in patients; induce redistribution, hyperproliferation, activation of NK and CD8+ T cells, and cytokine production.	189-191
			**The synergy of IL-15 and IL-21 in a mouse tumor model:** A cooperative effect on tumor regression, with apparent cures of large, established B16 melanomas; increase antigen-specific CD8+ T cell numbers.	
			**Co-administration of IL-15 with IL-12 and IL-18 in mice with myeloid leukemia:** Induce differentiation of cytokine-induced memory-like NK cells and thereby a vigorous anti-tumor response.	
			**CAR-T cells engineered to secrete IL-15:** Augment tumor cytotoxicity in vitro and in vivo, and increase T cell expansion on antigen recognition in vitro; provide long-term CAR-T cell-mediated immunity toward the cancer antigen along with enhancing CAR-T cell function within the TME.	
IL-18	Macrophages, DCs, and epithelial cells	Promote anti-tumor responses by stimulating IFN-γ production, activating NK cells and monocytes/macrophages, and enhancing the ADCC effects; enhance the differentiation of Th1 cells and facilitate the priming of effector cells.	**IL-18-based adoptive transfer (such as IL-18 preactivated NK cells):** ongoing clinical trials with promising results in AML patients.	192-195
			**Constitutively IL-18 secreting CAR-T cells:** Enhance CAR-T cell expansion and persistence, increase the survival of mice bearing CD19+ tumors, while also activating the endogenous immune system.	
			**CAR-T cells with inducible IL-18 secretion:** Reduce the size of advanced pancreatic tumors and prolong survival of mice; more effective at controlling late-stage disease than CAR-T cells producing IL-12.	
			**TCR transfer therapy with the inducible secretion of either IL-12 or IL-18:** IL-18 secretion by CAR-T cells could be more efficacious than IL-12 secretion and also safer for patient infusion.	

Notes: NHL, non-Hodgkin lymphoma; CR, complete response; ORR, overall response rate; CTL, cytotoxic T lymphocytes; HL, Hodgkin lymphoma; CTCL, cutaneous T cell lymphoma; R/R NHL, relapsed/refractory NHL.

**Table 3:** Platforms for engineering cells in vivo.

Platform	Engineered cells	Targeted receptor	Delivered plasmid	References
LV with NiV glycoproteins	Human T cells	Human CD8	hCD19-28ζ-CAR	169
LV with MV glycoproteins	Human T cells	Human CD4	hCD19-28ζ-CAR	196
LV with NiV glycoproteins	Human T cells	Human CD3	hCD19-28ζ-CAR	197
LV with SINV glycoproteins	Human T cells	Human CD3	hCD19-28ζ-CAR	198
AAV-DJ	Human T cells	N/A	hCD4-28-4-1BBζ-CAR	170
AAV-DJ	Mouse B cells	N/A	Cas9^gRNA^, anti-HIV bNAb	199
Synthetic DNA nanocarriers	Mouse T cells	Mouse CD3	mCD19-4-1BBζ-CAR	13
Synthetic mRNA nanocarriers	Human T cells	Human CD8	hCD19-28ζ-CAR, hROR1-4-1BBζ-CAR	171
LNP with DNA	Human T cells	Human CD3	hIL-6shRNA-anti-CD19 CAR	200
LNP with mRNA	Human T cells	Mouse CD5	mFAP-28ζ-CAR	168
Cavity-injectable nanoporter hydrogel with DNA	Mouse macrophages/microglia	N/A	hCD133-CD3ζ-CAR	175
Mannose-conjugated polyethyleneimine with DNA	Mouse M1 Macrophages	Mannose receptors	mALK-28ζ-CAR-IFN-γ	174

Notes: h, anti-human; m, anti-mouse; N/A, not applicable; LV, lentiviral vectors; AAV, adenovirus-associated vectors; bNAb, broadly neutralizing antibodies; MV, Measles virus; NiV, Nipah virus; SINV, Sindbis virus; LNP, lipid nanoparticle.

## Abbreviations

CAR: chimeric antigen receptor
TCR: T cell receptor
AML: acute myeloid leukemia
MM: multiple myeloma
AIDS: acquired immune deficiency syndrome
SLE: systemic lupus erythematosus
TME: tumor microenvironment
CRS: cytokine release syndrome
ITAM: immunoreceptor tyrosine-based activation motif
scFv: antibody single-chain variable region fragment
MHC: major histocompatibility complex
MR1: MHC-related protein 1
LFA-1: lymphocyte function-associated antigen 1
VHH: variable domain on a heavy chain
CDR3: complementary-determining region 3
CIK: cytokine-induced killer
TRUCK: T cells redirected for universal cytokine killing
SUPRA: Split, universal, and programmable
BiTE: bispecific T cell engager
TAC: T cell antigen coupler
TruC: T cell receptor fusion construct
STAR: synthetic T cell receptor and antigen receptor
DCV: dense core vesicle
GVHD: graft-versus-host disease
iNKT: invariant natural killer T
ADCC: antibody-dependent cell-mediated cytotoxicity
iPSC: induced pluripotent stem cell
TIR: intracellular toll/IL-1R
Treg: regulatory T cell
TNP: 2,4,6-trinitrophenol
CEA: carcinoembryonic antigen
TAN: tumor-associated neutrophil
hPSC: human pluripotent stem cell
GBM: glioblastoma
ROS: reactive oxygen species
RSV: respiratory syncytial virus
BCR: B cell receptor
MSC: mesenchymal stem cell
FITC: fluorescein isothiocyanate
cp-Fab: chemically programmed antibody fragment
FKBP12: FK506-binding protein 12
FRB: FKBP12-rapamycin binding domain
mTOR: mammalian target of rapamycin
PROTAC: protein hydrolysis targeted chimera
VIPER: versatile protease regulatable
BD: bromodomain
HCV-NS3: hepatitis C virus NS3 protease
ODD: oxygen-dependent degradation domain
HIF: hypoxia-inducible factor
ZFP-VP64: zinc finger protein-VP64
HSV: herpes simplex virus
HiTA: hypoxia-inducible transcription amplification
LiCAR: light-switchable CAR
Hsp: heat shock protein promoter
PGK: phosphoglycerate kinase 1 promoter
FUS: focused ultrasound
iRC9, rapamycin-induced: caspase9-based suicide switch
GCV: Ganciclovir
tEGFR: truncated form of epidermal growth factor receptor
SynNotch: Notch-based assembled receptor
CCR: chimeric costimulatory receptor
SEAKER: synthetic enzyme-armed killer
LNP: lipid nanoparticle
